# Mapping the local effectiveness of mass drug administration for malaria using transportability methods

**DOI:** 10.1038/s44360-026-00094-1

**Published:** 2026-03-24

**Authors:** Michelle E. Roh, Yanwei Tong, Gabriella Barratt Heitmann, Junran Jia, El-hadji Konko Ciré Ba, Jean Louis Ndiaye, Ari Fogelson, Paul Milligan, Amadou Seck, Abdoulaye Diallo, Aminata Colle Lo, Michael Baiocchi, Roly Gosling, Adam Bennett, Michelle S. Hsiang, Jade Benjamin-Chung

**Affiliations:** 1 Institute for Global Health Sciences, University of California, San Francisco (UCSF), San Francisco, CA, USA.; 2 Department of Obstetrics and Gynecology, Oregon Health and Science University, Portland, OR, USA.; 3 Department of Epidemiology and Population Health, Stanford University, Stanford, CA, USA.; 4 Department of Statistics, Stanford University, Stanford, CA, USA.; 5 Université Iba Der Thiam de Thiès, Thiès, Senegal.; 6 Department of Infectious Disease Epidemiology, London School of Hygiene and Tropical Medicine, London, UK.; 7 Department of Disease Control, London School of Hygiene and Tropical Medicine, London, UK.; 8 PATH, Seattle, WA, USA.; 9 Department of Epidemiology and Biostatistics, UCSF, San Francisco, CA, USA.; 10 Department of Pediatrics, UCSF, San Francisco, CA, USA.; 11 Chan Zuckerberg Biohub, San Francisco, CA, USA.; 12 Deceased: Jean Louis Ndiaye.

## Abstract

Numerous trials have evaluated the effectiveness of mass drug administration (MDA) in rapidly reducing malaria transmission, but it is unknown whether the estimated effects generalize to other populations eligible for MDA. A recent cluster randomized trial in Senegal found that MDA reduced malaria incidence by 55% in areas that routinely deploy seasonal malaria chemoprevention (SMC). Here, we used transportability models with machine learning to generalize trial effects to 116 non-trial communes where SMC is the standard of care. Accounting for differences in weather, vegetation and population density between trial and non-trial areas, we estimated considerable reductions in incidence (ranging from 36% to 65%) in 74 non-trial communes, with larger decreases in areas having higher precipitation, denser vegetation and lower temperatures. We found that MDA was not effective in the postintervention year in non-trial communes, supporting the notion that MDA’s effects are short-lived. Our approach offers a scalable framework for generalizing trial findings to target environmentally mediated infectious disease interventions.

Malaria remains a major global public health burden, with an estimated 263 million cases and 597,000 attributable deaths in 2023 (ref. 1). Despite considerable advances in reducing the disease burden, progress towards malaria elimination has stalled. This stagnation has been particularly pronounced in countries with moderate to high transmission^1^, where more intensive or new strategies may be required to accelerate progress.

Mass drug administration (MDA) has regained attention as a potential strategy to accelerate progress towards malaria elimination. MDA involves administering a full treatment course of antimalarials to all individuals within a defined geographical area, regardless of their infection status, with the frequency and duration of rounds tailored to the intervention’s goal—that is, to either rapidly reduce the burden or interrupt transmission. To date, 12 cluster randomized controlled trials have evaluated MDA for transmission reduction^2-4^, including 4 trials conducted in moderate to high transmission settings^3-6^. A recent meta-analysis of ten of these trials^2^ reported mixed findings regarding the effectiveness of MDA, with most reporting only short-term reductions in incidence and/or prevalence in very low to low transmission settings^2-4^. Consistent with these findings, mathematical models also suggest that the effects of MDA are probably time-limited and highly context-dependent^7^. As such, the World Health Organization (WHO) recommends MDA for very low to low transmission settings but not for moderate or high transmission settings due to low certainty of evidence^8^.

Although numerous cluster randomized trials and meta-analyses have evaluated the effectiveness of MDA^2,9^, the generalizability of their results to broader settings remains unclear. While cluster randomized trials are inherently designed to yield internally valid estimates (by minimizing confounding through randomization), they may offer limited external validity for two key reasons. First, cluster randomized trials typically estimate treatment effects as the average differences between intervention and control clusters. This approach may obscure meaningful heterogeneity in intervention impact, especially when contextual factors such as transmission intensity, vector ecology or human mobility vary across clusters^10-12^. Second, cluster randomized trials often include a limited number of clusters and impose strict eligibility criteria to facilitate operational feasibility, enrolment, statistical power, intervention delivery and follow-up^13^, which can constrain generalizability. These limitations carry over to meta-analyses, which pool data from numerous trials to increase statistical power. However, most of these trials are conducted in a limited number of settings and may not represent the target populations to which the interventions would be applied. As a result, the pooled effect estimates, which form the basis for WHO policies, may have low external validity. Because MDA’s effectiveness may be highly context-dependent, understanding the drivers of heterogeneity in effectiveness is critical to identifying key areas where MDA could be targeted to maximize its cost-effectiveness, a priority outlined by the WHO Global Technical Strategy for Malaria 2016–2030 (ref. 14).

In recent years, transportability models have been developed in the causal inference literature to assess the external validity of studies by extrapolating intervention effect estimates from trials to external target populations^15^. These models require data on factors that modify the intervention effect (that is, ‘effect modifiers’) and differ in distribution between trial and target populations^16^. A key advantage of this approach is that it does not strictly require outcome data (for example, malaria incidence or prevalence) from the external target population. Instead, transportability models use information on the distribution of effect modifiers in both the study and target (non-trial) populations to estimate intervention effects beyond the original study setting. For environmentally mediated infections such as malaria, fine-scale remote sensing data have immense potential as a source of effect modifier data in transportability analyses. Moreover, remote sensing data are widely available, typically free of charge, cover large geographical areas, and offer high spatial and temporal resolution, making them well suited for transporting intervention effects across entire countries and regions.

Transportability models are complementary to mathematical models, which the WHO recommends for informing data-driven subnational targeting of interventions^14^. Mathematical models integrate a mechanistic understanding of transmission dynamics to simulate the potential impacts of interventions under various scenarios. However, they often rely on strong assumptions and parameter inputs that are difficult to validate and may not accurately reflect local conditions^17^. Further, competing models often produce substantially different intervention effect estimates^7^. As a result, the findings might not hold in populations with differing epidemiological or entomological characteristics^18^. Conversely, transportability analyses leverage real-world trial data and localized effect modifier data to estimate intervention effects in new populations, thereby providing an additional framework to inform the targeting and tailoring of interventions for maximum benefit.

Here, we transport the effects of MDA estimated from a recent cluster randomized trial in Senegal^4^ to non-trial areas of Senegal where seasonal malaria chemoprevention (SMC) is offered as the standard of care. Our goal was to characterize geographical areas where MDA may be more or less effective, accounting for differences in environmental and demographic variables.

## Results

### Original trial results

**Fig. 1 ∣:**
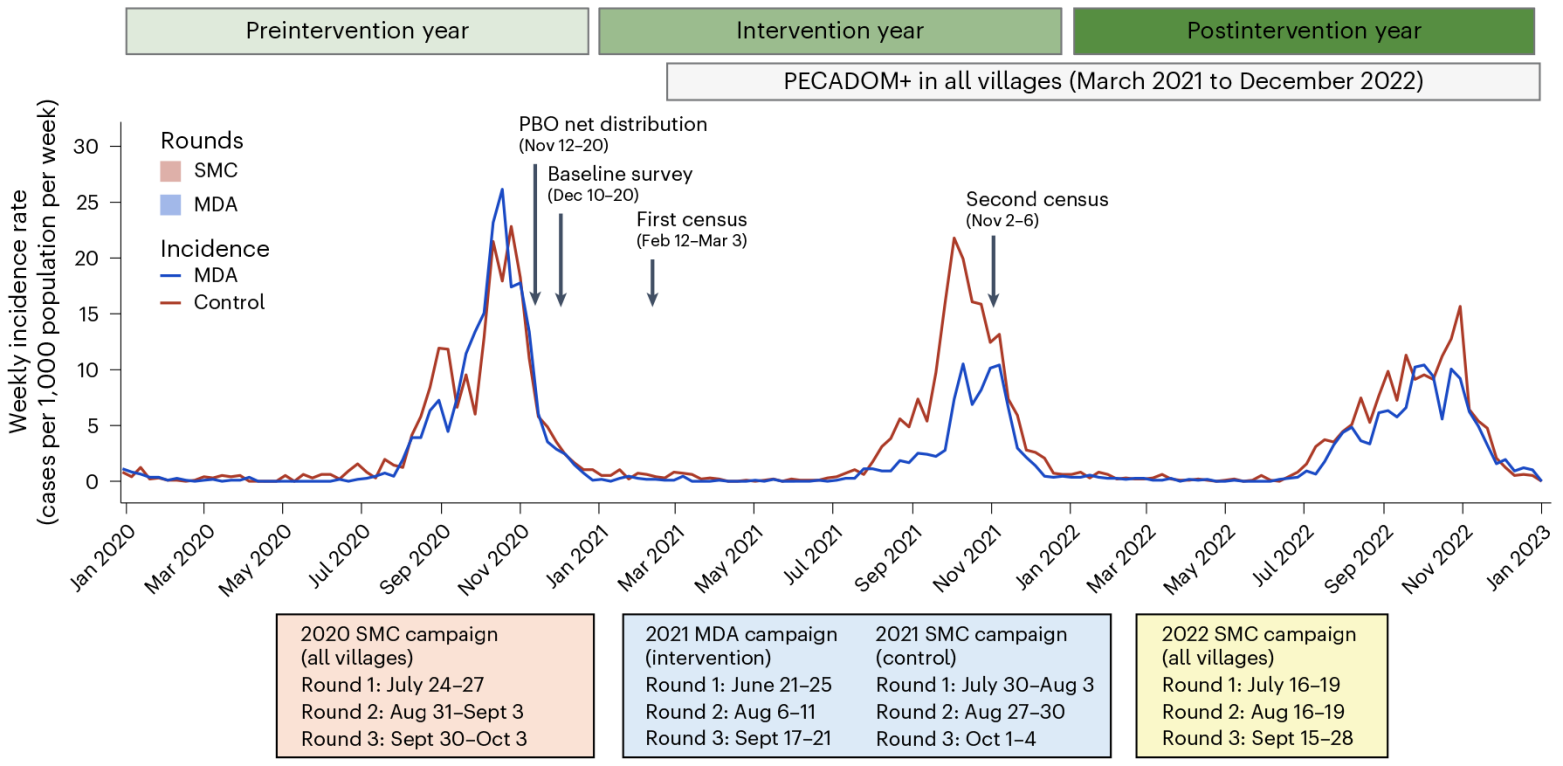
Intervention distribution and weekly malaria incidence over time during the preintervention, intervention and postintervention years. Weekly malaria incidence rates (cases per 1,000 population per week) in mass drug administration (MDA) intervention and control villages, January 2020–January 2023. Vertical bars indicate timing of seasonal malaria chemoprevention (SMC) rounds (all villages) and MDA rounds (intervention villages only). Arrows denote key study milestones including PBO net distribution, baseline survey and census rounds.

We conducted a secondary analysis of a two-arm, cluster randomized controlled trial evaluating the impact of MDA on malaria transmission (NCT04864444)^4^. The trial was conducted from September 2020 to December 2022 in a low to moderate transmission setting in southeastern Senegal. Sixty villages were assigned to receive either the intervention or control using stratified, constrained randomization. In intervention villages, individuals aged ≥3 months received three rounds of MDA with dihydroartemisinin–piperaquine plus a single low dose of primaquine every 6 weeks, starting on 21 June 2021—approximately 1 month before the transmission season (July–December)—to clear the parasite reservoir. In control villages, children aged 3–120 months received standard-of-care SMC with sulfadoxine–pyrimethamine plus amodiaquine every 4 weeks, starting at the presumed beginning of the transmission season (30 July 2021). MDA was implemented for 1 year (2021), after which all villages resumed SMC in 2022. Before the intervention, all villages received pyrethroid–piperonyl butoxide (PBO) bed nets at high coverage (~98%)^4^. The timeline of study activities is shown in Fig. 1.

During the intervention year (2021), environmental variables and travel time to the nearest health facility were well balanced between the arms (Supplementary Appendix 1). Population density was higher in control villages than in those receiving MDA. Median village-level coverage was 68%, 73% and 74% across the three MDA rounds, and 93%, 92% and 92% across the three SMC rounds. In the original trial, MDA reduced clinical malaria incidence by 55% (95% confidence interval (CI): 28%, 71%) during the transmission season of the intervention year (July–December 2021) and by 26% (95% CI: −17%, 53%) in the following transmission season (July–December 2022), when all villages resumed SMC^4^.

### Effect heterogeneity within trial clusters

**Fig. 2 ∣:**
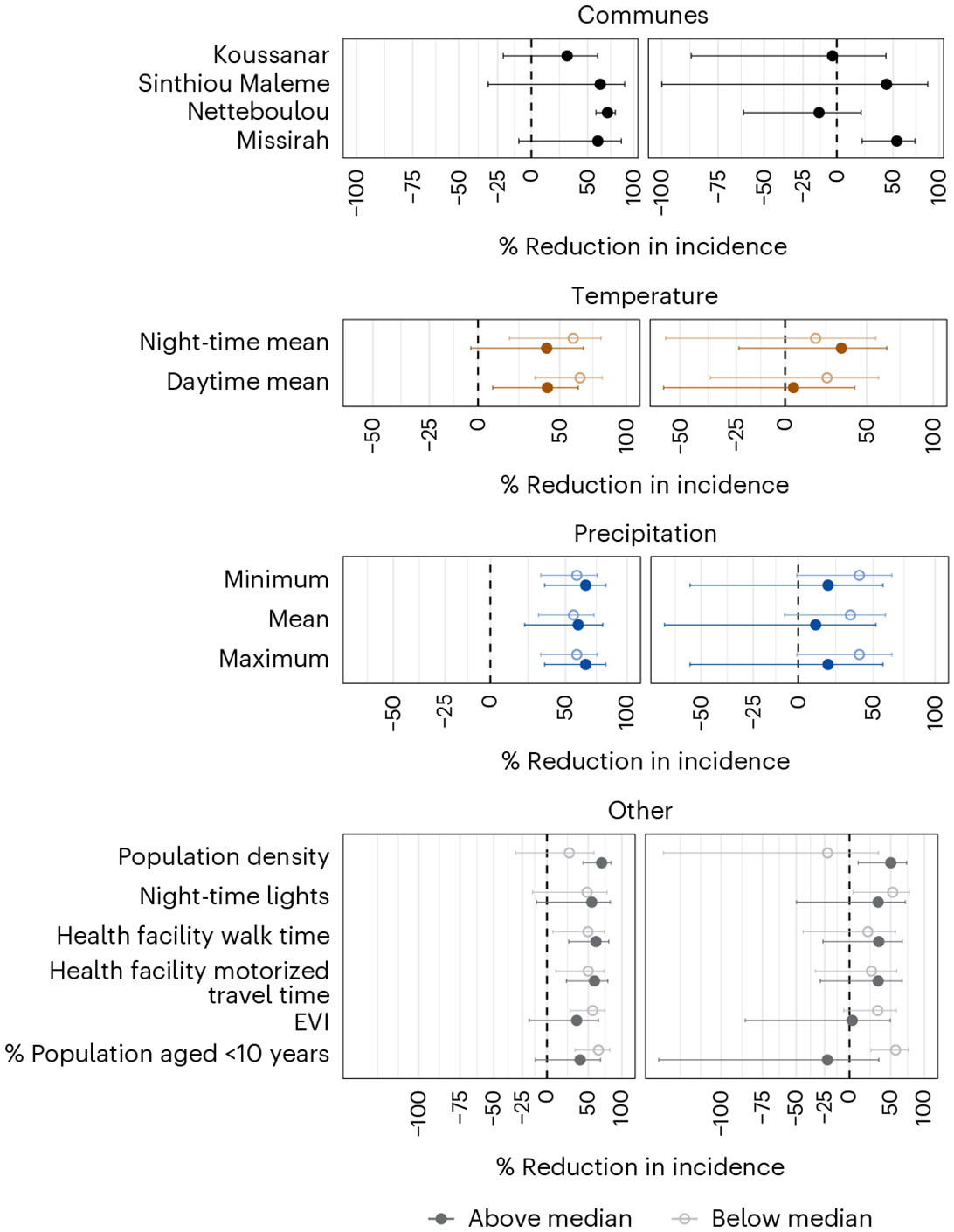
Heterogeneous effects of MDA during the intervention and postintervention years. Point estimates represent adjusted incidence ratios from subgroup analyses comparing the impact of MDA on malaria incidence during the intervention year (left) and the postintervention year (right) across potential spatial, environmental and demographic effect modifiers. For commune subgroup analyses, incidence ratios compare the incidence between the MDA arm and the control arm. Other effect modifiers were coded as binary indicators for values above or below the median in a given year. Analyses were performed at the cluster level. The analysis dataset included the preintervention, intervention and postintervention years (*n* = 3 years), with 60 clusters (30 per arm) per year (*n* = 180 clusters in total). Horizontal bars indicate 95% CIs computed using robust standard errors. In the postintervention year, the lower bound for Sinthiou Maleme was imputed for clarity of visualization; the estimated value was −104%. Incidence ratios were adjusted for the timing of case detection relative to the full PECADOM+ scale-up, as in the original trial analysis.

To identify candidate effect modifiers for transportability analyses, we assessed whether MDA’s effects varied by spatial, environmental and demographic factors (Fig. 2). Effect modification was assessed on both the multiplicative and additive scales (Supplementary Appendixes 2-4)^19^. Spatial variation was assessed by estimating effects at the commune level—the smallest standard administrative unit used for public planning in Senegal and the scale at which trial estimates were transported. Environmental variables were lagged by 1–3 months to account for delayed impacts on vector breeding, survival and biting.

Across the four communes that encompassed trial villages (Koussanar, Sinthiou Maleme, Netteboulou and Missirah), we observed substantial heterogeneity in MDA’s effects (Fig. 2 and Supplementary Appendix 2). During the intervention year (2021), the effect sizes in the three southernmost communes (Sinthiou Maleme, Netteboulou and Missirah) were greater than the overall effect estimate in the original trial (percentage reduction: 55% (95% CI: 28%, 71%)^4^). In Netteboulou, the percentage reduction in malaria incidence was 70% (95% CI: 58%, 79%). In Sinthiou Maleme and Missirah, the estimated impact of MDA was a reduction of approximately 60%, although the 95% CIs included the null. In the postintervention year (2022), MDA did not have a statistically significant impact on incidence (similar to the original trial estimate), except in the southernmost commune of Missirah (54% (95% CI: 21%, 73%) versus 26% (95% CI: −17%, 53%)^4^). Tests of interaction between commune and treatment arm did not reach the 0.05 threshold for statistical significance on either the ratio or additive scales in 2021 (Supplementary Appendix 3). In 2022, evidence of interaction was found on the ratio scale in Netteboulou and Missirah and on the additive scale in Missirah (Supplementary Appendix 4).

Next, we linked village centroids to publicly available remote sensing data to examine heterogeneity in intervention effects by temperature, precipitation, enhanced vegetation index (EVI), population density, night-time lights and travel time to the nearest health facility. These variables were selected a priori based on the hypothesis that they may modify MDA’s effectiveness: environmental conditions are known to influence malaria transmission dynamics; travel time to a health facility may serve as a proxy for MDA coverage in real-world implementation settings; and night-time light radiance may reflect underlying household socioeconomic status. For each variable, we conducted stratified analyses based on cluster values that fell above or below the median (Fig. 2 and Supplementary Appendix 2). During the intervention year, MDA was more effective in areas with below-median mean temperature levels (additive scale interaction *P* = 0.072 for mean daytime temperature and 0.107 for mean night-time temperature) (Supplementary Appendix 3). In both the intervention and postintervention years, MDA was more effective in areas with above-median population density (ratio scale interaction *P* = 0.020 in 2021 and 0.091 in 2022) and below-median proportion of the population aged under 10 years (ratio scale interaction *P* = 0.048 in 2021 and 0.008 in 2022) (Supplementary Appendixes 3 and 4).

### Heterogeneity of environmental and demographic characteristics across Senegal

**Fig. 3 ∣:**
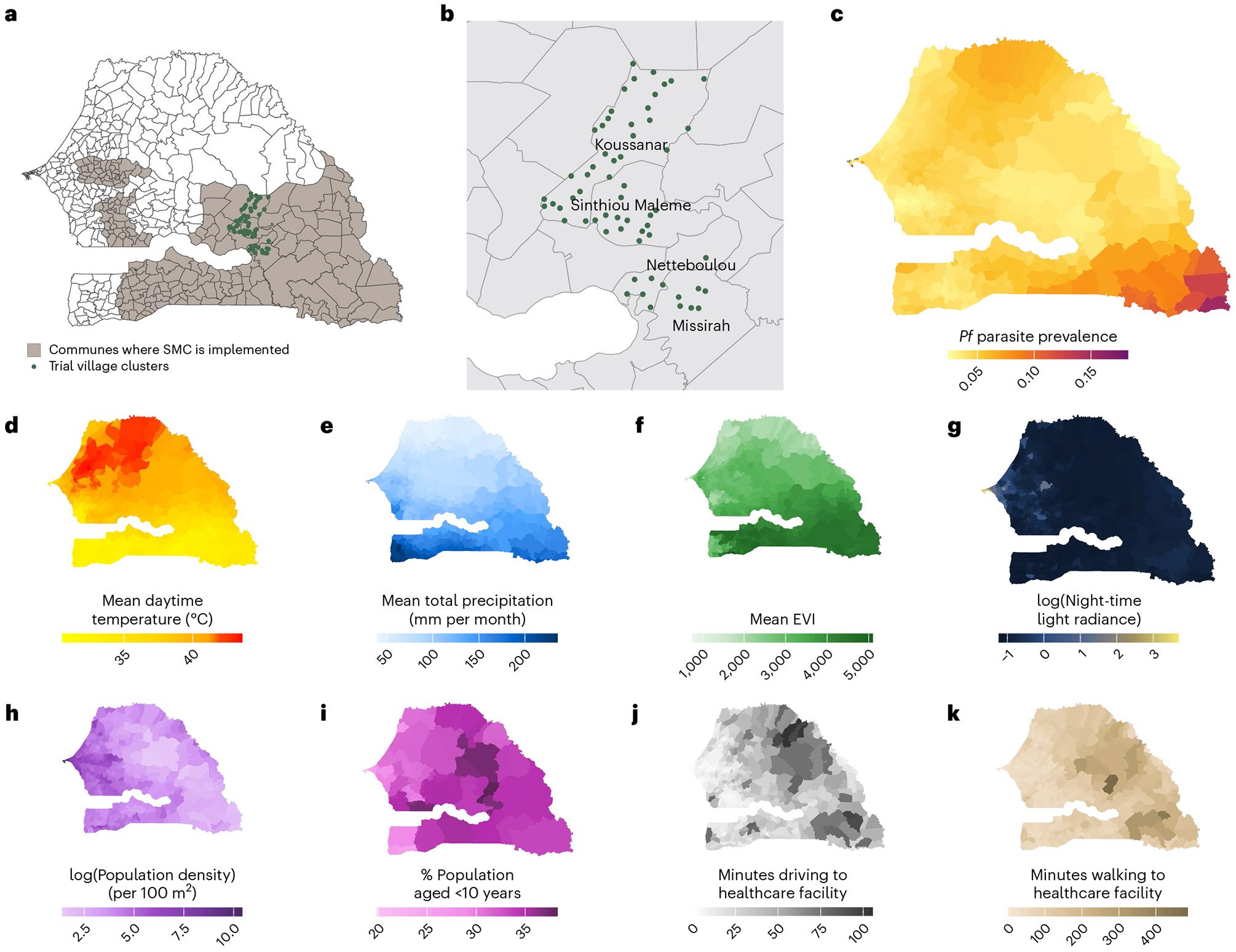
Maps depicting areas routinely implementing SMC, the locations of trial villages, and variations in environmental and demographic covariates. **a**, Areas where SMC was routinely implemented in Senegal during the trial period (grey-shaded areas) and the 60 clusters included in the original trial (green points). **b**, Trial villages (green points) nested within the four communes encompassing the study site. **c**, Predicted prevalence of *P. falciparum* (*Pf*) malaria in 2021–2022, extracted from the Malaria Atlas Project. **d**–**k**, Variables used to assess effect heterogeneity, including environmental variables (**d**–**f**), demographic variables (**g**–**i**) and travel time (in minutes) by driving (**j**) or walking (**k**) to the nearest health facility. Values are shown as the mean for each commune during the July–December transmission seasons of 2021 and 2022. Basemap from GADM (https://gadm.org/).

To assess the potential for transporting trial findings across Senegal, we examined the spatial heterogeneity of potential effect modifiers at the national level. For each commune, we mapped mean values during the 2021 and 2022 transmission seasons (Fig. 3). Communes where SMC was routinely implemented had lower mean temperatures, night-time light radiance and population density, but higher total precipitation and EVI, compared with communes where SMC was not implemented. Within SMC-implementing areas, population density exhibited the greatest spatial variability between communes (coefficient of variation (CV) = 109). CVs for motorized travel time to the nearest health facility, night-time lights, walking time to the nearest health facility, mean precipitation, EVI, daytime temperature and the proportion of the population aged <10 years were 70, 67, 65, 26, 18, 9 and 6, respectively. The extent to which the values of effect modifiers at the trial site were similar to those at non-trial sites varied by follow-up month. For example, EVI and precipitation were higher at the trial site than at most non-trial sites from July to September, but lower during other months (Supplementary Appendix 5).

### Transporting MDA’s effects to non-trial areas

**Fig. 4 ∣:**
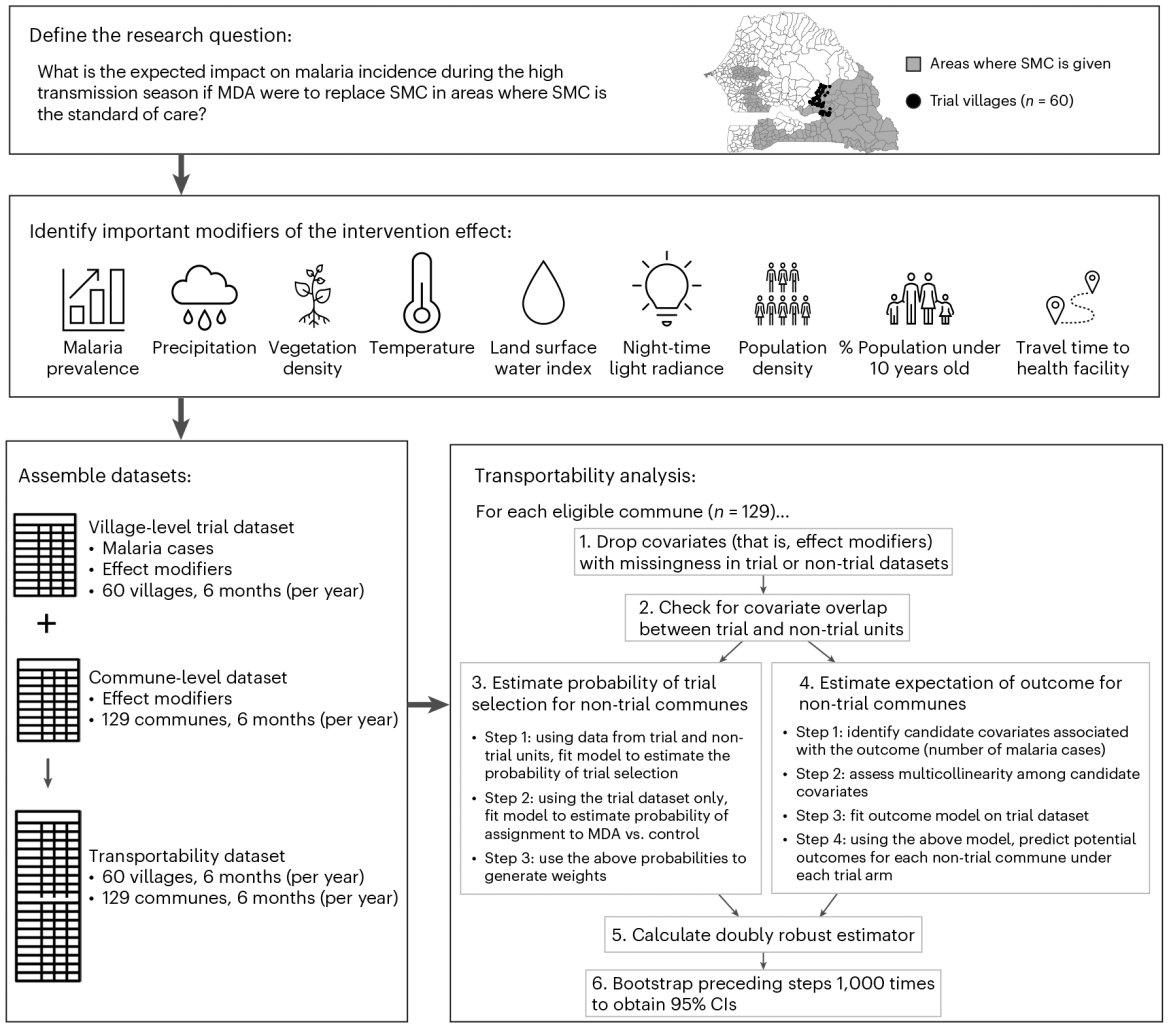
Schematic framework of transportability analyses. Basemap from GADM (https://gadm.org/). Spreadsheet icon from Selman Design via icon-icons.com under a Creative Commons license CC BY 4.0.

To estimate the impact of MDA in areas beyond the trial’s geographical footprint, we conducted transportability analyses using a doubly robust modelling approach^20^. This method treats trial clusters as a sample of a broader target population, defined here as communes in Senegal where SMC is implemented as the standard of care. A schematic overview of the transportability framework is shown in Fig. 4. Because the trial was conducted under conditions of high vector control coverage, prompt malaria case management and robust surveillance, the transported effect estimates assume comparable programmatic conditions between the trial and non-trial areas.

Of the 433 communes in Senegal, 129 were eligible for transportability analyses (Supplementary Appendix 6). Of these, four communes (Koussanar, Sinthiou Maleme, Netteboulou and Missirah) included trial villages. Communes were excluded if SMC was not implemented as the standard of care (*n* = 247) and/or if the population density exceeded that of the trial site (>152 people per 100 m^2^) (*n* = 57). The distributions of most potential effect modifiers overlapped between the trial clusters and the 129 eligible non-trial communes (Supplementary Appendix 7). The effect modifiers included in the transportability models were precipitation, temperature, EVI and population age distribution (see details in the Methods).

Our doubly robust modelling approach relied on two components. First, for each commune, we fitted a model to estimate the probability that the commune would have been included in the trial at month *t* based on its effect modifier values. The predicted probabilities for each commune were skewed and right-modal, suggesting that the communes were probably representative of the target population when accounting for differences in effect modifiers (Supplementary Appendix 8). There was substantial overlap in the probabilities between trial and non-trial areas for most communes, which is necessary for causal identification in transportability analyses. Next, we modelled the expected malaria incidence in each commune at month *t* as a function of effect modifiers using trial data, then predicted outcomes in each arm in non-trial communes. We then implemented a doubly robust estimator that combines both approaches and is consistent if either the outcome or selection model is correctly specified^20^. For each SMC-implementing commune, we estimated MDA’s effectiveness as (1 – incidence ratio) × 100%. We performed separate transportability analyses for the intervention and postintervention years. Outcome models used for transportability did not account for baseline malaria incidence or include a population offset, as was done in the original trial, because such data were not available in non-trial areas. Comparison of estimates from transportability models and the original trial showed minimal differences in point estimates and 95% CIs during the intervention year, and moderate differences in the postintervention year; however, the interpretation of findings was similar between approaches, with overlapping 95% CIs (Supplementary Appendixes 9 and 10).

### Estimated effects of MDA in non-trial areas

**Extended Data Fig. 1 ∣:**
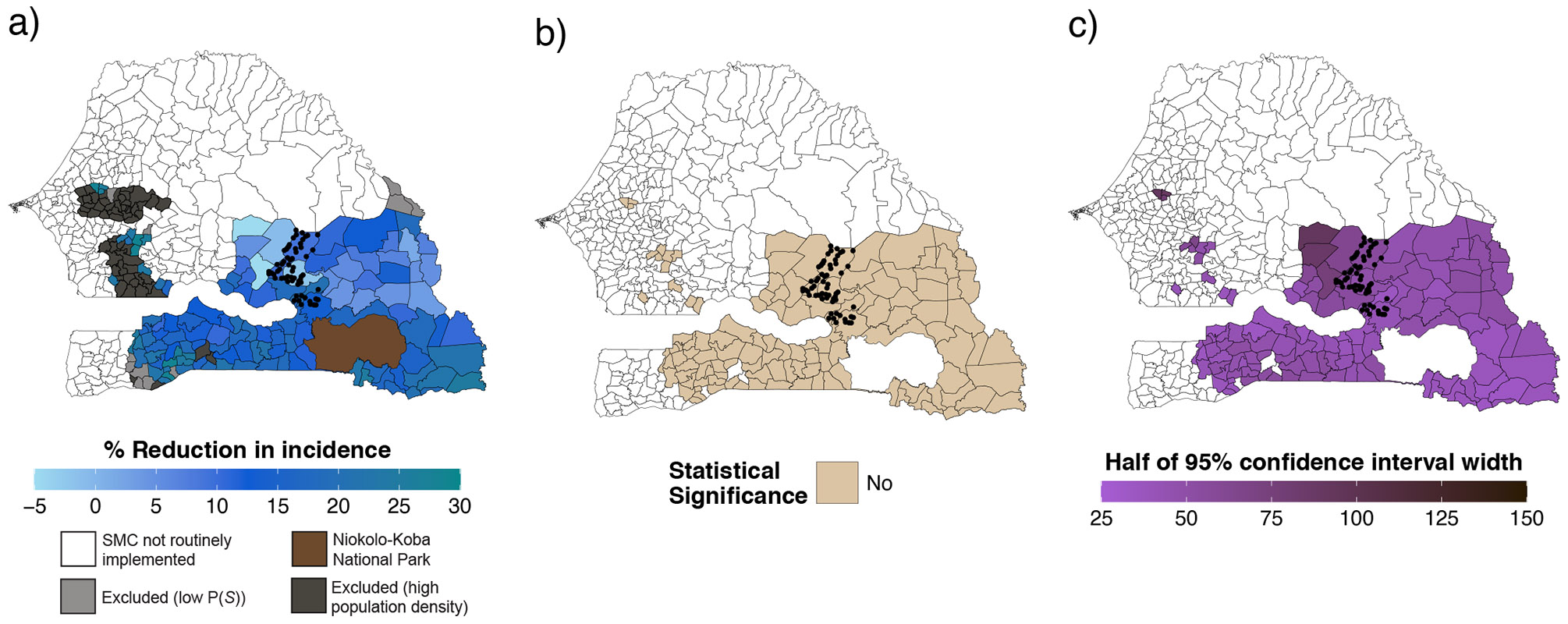
Spatial distribution of transported effects of mass drug administration in the post-intervention year (2022). Polygons indicate communes. Panel (**a**) shows the percent reduction in malaria, expressed as 1 - the ratio of cases in the MDA vs. control arm during the transmission season of the post-intervention year (2022), estimated using doubly robust transportability models. Covariates included in transportability models included precipitation, temperature, enhanced vegetation index, and population density. The final covariate included in transportability models varied between communes following screening for collinearity, data sparsity, association with the malaria case count, and feature selection using elastic net regression. Transportability analyses were restricted to communes where SMC was routinely offered during the trial period, had a population size <152 per km, and a predicted probability of trial selection (P(*S*)) of >0.75. Separate analyses were performed for each Commune-month (N = 366 per Commune). Panel (**b**) indicates whether the 95% confidence interval for transported estimates for each commune included the null. Panel (**c**) depicts uncertainty, shown as half the length of the 95% confidence interval, estimated using a non-parametric bootstrapping procedure that resampled commune-months within trial datasets and months within the non-trial commune 1,000 times with replacement.

**Fig. 5 ∣:**
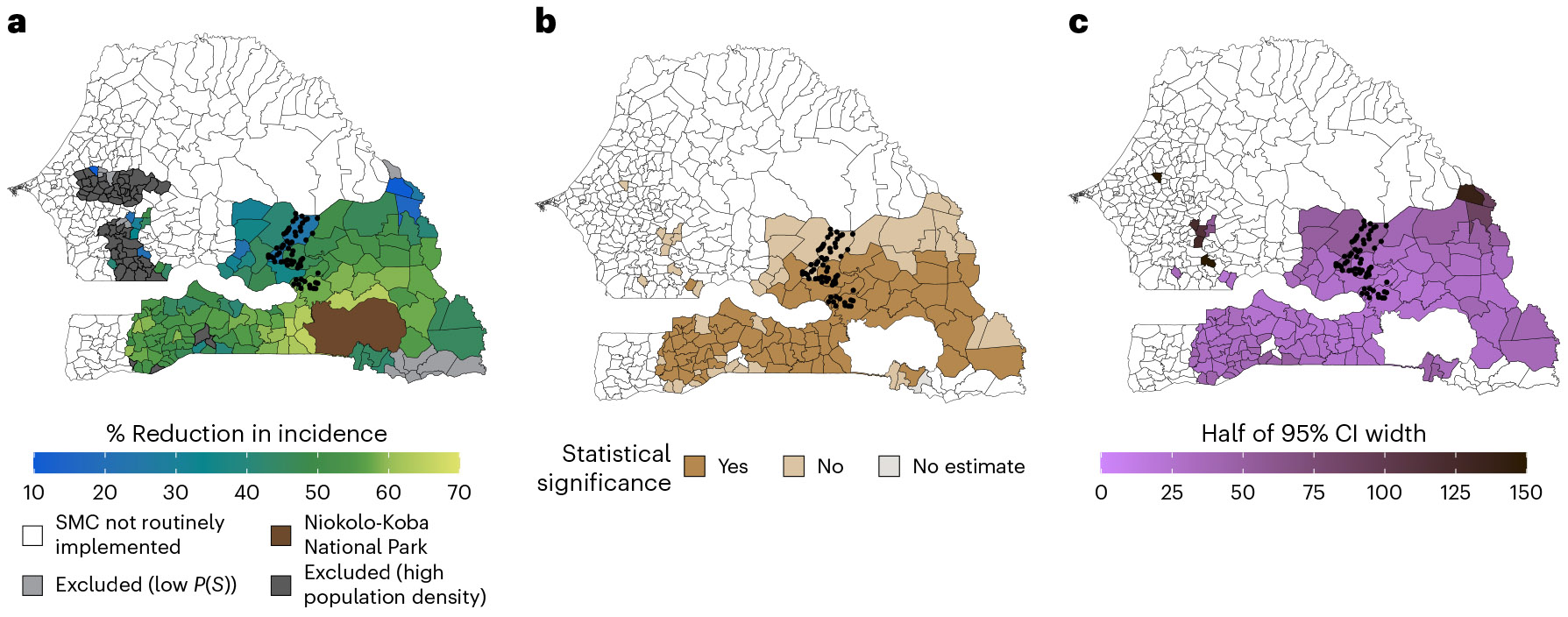
Spatial distribution of the transported effects of MDA. **a**, Percentage reduction in malaria incidence (1 – the ratio of cases in the MDA versus control arm) during the transmission season of the intervention year (2021), estimated using doubly robust transportability models. Covariates included precipitation, temperature, EVI, population density and the percentage of the population <10 years old. The covariate list included in the final models varied for each commune-month, following screening for collinearity, data sparsity, association with the malaria case count and feature selection using elastic net regression. Transportability analyses were restricted to communes where SMC was routinely offered during the trial period, that had a population size of <152 people per 100 m^2^ and that had a predicted probability of trial selection (*P*(*S*)) of >0.75. Separate analyses were performed for each commune-month (*n* = 366 per commune). **b**, Map indicating whether the 95% CIs for transported estimates include the null. **c**, Uncertainty—depicted as half the width of the 95% CI—estimated using a non-parametric bootstrapping procedure that resampled commune-months within trial datasets and months within the non-trial commune 1,000 times with replacement. Polygons indicate communes. Boundaries for Niokolo-Koba National Park were obtained from the World Database on Protected Areas via Protected Planet (www.protectedplanet.net). Basemap from GADM (https://gadm.org/).

**Fig. 6 ∣:**
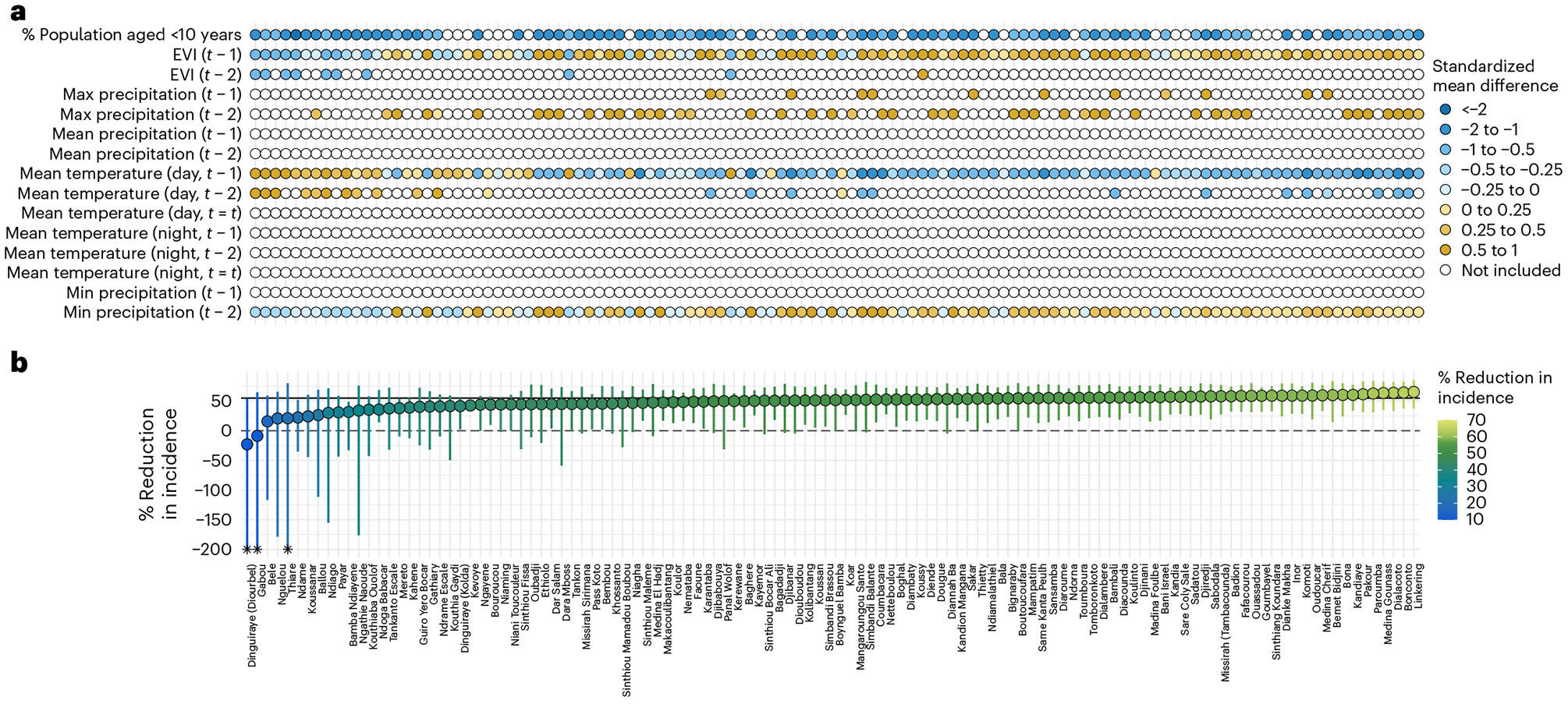
Transported effect estimates of MDA to non-trial areas during the intervention year, including covariates used in the transportability analysis. **a**, Covariates used in the transportability analysis for each commune. Coloured points indicate covariates included in the transportability models after screening for collinearity, data sparsity, association with malaria case counts and feature selection using elastic net regression. Blue- and yellow-shaded points indicate the standardized mean difference for a given covariate in a given commune, calculated as the mean in a non-trial commune minus the mean in the trial site divided by the pooled s.d. White points indicate covariates that did not pass the screening. **b**, Transported effect for each commune (coloured points), expressed as the percentage reduction in malaria incidence (1 – the ratio of cases in the MDA versus control arm) during the transmission season of the intervention year, using doubly robust transportability models. Analyses were restricted to communes with a population size of ≤152 people per 100 m^2^, those with a predicted probability of trial participation of >0.75, and communes where SMC was offered during the trial period. Separate analyses were performed for each commune using monthly data from July to December (*n* = 366 per commune). Analyses used doubly robust transportability models. Vertical coloured lines indicate the 95% CI for each commune, obtained from a non-parametric bootstrap that resampled commune-months 1,000 times with replacement. Communes are sorted according to the transported effect size. The grey solid line indicates the original trial estimate (55%; 95% CI: 28%, 71%). Asterisks indicate CIs that were truncated for data visualization (lower bound < −200% and/or upper bound > 100%).

Of 129 eligible communes, we were able to transport estimates for 116 during the intervention year (Fig. 5a) and for 118 in the postintervention year (Extended Data Fig. 1). Estimates were not transported in 13 communes during the intervention year and in 11 communes during the postintervention year due to potential positivity violations (that is, probability of trial participation < 0.75). During the intervention year, the predicted effectiveness of MDA varied across communes, ranging from −23% to 65% (Figs. 5 and 6). In 74 communes (64%), we estimated statistically significant reductions ranging from 36% to 65%, with estimates exceeding the original trial estimate in 37 communes (32%)—ranging from 56% to 65%. The effect size and precision of estimates, defined by the width of 95% CIs, were generally higher in communes located in the southeastern region of Senegal, where environmental and demographic characteristics were more similar to those of the trial site (Fig. 5c). In the postintervention year, most transported effects were closer to the null compared with the original trial estimate (26% (95% CI: −18%, 53%)^4^), with none reaching statistical significance (Extended Data Fig. 1).

### Drivers of heterogeneity in transported effects

**Extended Data Fig. 2 ∣:**
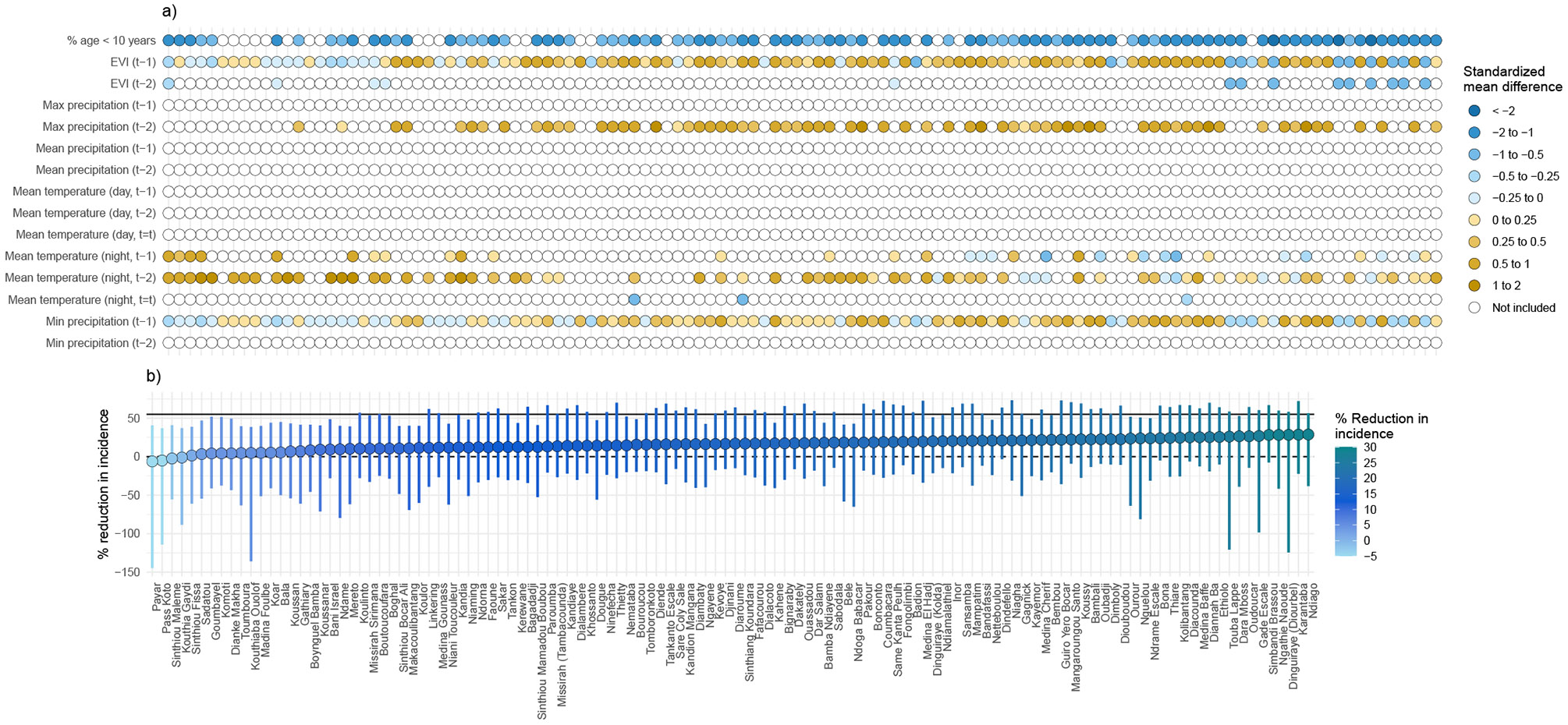
Transported effect estimates of mass drug administration to non-trial areas during the post-intervention year (2022) and covariates used in transportability analysis. Panel (**a**) depicts covariates used in the transportability analysis for each commune. Colored points indicate covariates included in the transportability models after screening for collinearity, data sparsity, association with the malaria case count, and feature selection using elastic net regression. Blue and yellow shaded points indicate the standardized mean difference for a given covariate in a given commune, calculated as the mean difference between the non-trial commune and trial site divided by the pooled standard deviation. White points indicate covariates that did not pass screening. In Panel (**b**), colored points indicate transported effect for each Commune, expressed as the percent reduction in malaria incidence (1 – the ratio of cases in the MDA vs. control arm) in the post-intervention year (2022). Colored vertical lines indicate 95% confidence interval for each commune, which was obtained from non-parametric bootstrap that resampled commune-months within trial datasets and months within non-trial areas for a given commune 1,000 times with replacement. Transportability analyses were performed separately for each commune using monthly data from July to December (N = 366 per commune). Transportability analyses restricted to communes with population size ≤152 per km, a predicted probability of trial participation >0.75, and where SMC was offered during the trial period.

To explore the potential drivers of heterogeneity in transported effect estimates, we calculated the standardized mean difference for effect modifiers between each commune and the trial site. During the intervention year, communes with larger reductions in incidence for MDA versus control tended to have higher EVI and precipitation levels, while having lower mean temperatures and a lower percentage of children under 10 years old, compared with communes with smaller reductions (Fig. 6). In the postintervention year, there appeared to be slightly larger reductions in communes with a lower percentage of children under 10 years old, but the magnitude of these reductions was small and did not reach statistical significance (Extended Data Fig. 2).

### Sensitivity analyses

To validate our transportability model, we transported trial estimates to areas surrounding the trial villages (Supplementary Appendix 11), where effect modifier values are expected to be similar; therefore, the transported estimates were expected to align closely with the original trial results. In the intervention year (2021), the transported percentage reduction in malaria incidence at the trial site was 50% (95% CI: 18%, 71%) compared with 55% (95% CI: 28%, 71%) in the original trial and 49% (95% CI: 25%, 66%) using trial data without adjustment for baseline malaria incidence (SupplementaryAppendix 9)^4^. In the postintervention year (2022), the transported percentage reduction at the trial site was 4% (95% CI: −64%, 33%) compared with 26% (95% CI: −17%, 53%) in the original trial^4^ and 4% (95% CI: −28%, 29%) using trial data without baseline adjustment (Supplementary Appendix 9).

Next, MDA coverage in the original trial varied across intervention clusters (range, 54–80%), which may have influenced our transported estimates. To assess whether some of this variation was captured by effect modifiers considered in our transportability models, we examined the correlations between coverage and these effect modifiers. Correlations were generally weak (∣*ρ*∣ < 0.2) for most modifiers, except for daytime temperature, precipitation and travel time to the nearest health facility, which demonstrated moderate to high correlations with coverage (Supplementary Appendix 12).

## Discussion

In this re-analysis of a cluster randomized trial from Senegal, we generalized trial-based estimates to other parts of the country with the goal of identifying areas where MDA could be integrated with existing interventions to rapidly reduce the malaria burden. Within the trial, we found substantial spatial heterogeneity in the effectiveness of MDA, with the greatest impacts observed in the southernmost communes. In the intervention year, we estimated that MDA would have reduced malaria incidence by 36–65% in 64% of eligible non-trial communes. Notably, the estimated effects exceeded the original trial’s overall estimate in 32% of communes during the intervention year, mainly in regions just south of the trial site, which had higher precipitation, denser vegetation and lower temperatures. However, the effects were not sustained following the discontinuation of MDA in any of the non-trial communes, aligning with conclusions from prior studies and WHO recommendations^8^ that MDA can have a strong but transient effect on malaria incidence.

Here, we applied transportability methods to generalize estimates from a large, population-level malaria intervention trial. By integrating trial data with fine-scale remote sensing, this approach offers a powerful, data-driven framework to identify areas where interventions would yield the greatest impact, making it a valuable addition to the existing toolbox for guiding subnational targeting. For MDA and other aggressive malaria interventions, such targeted implementation is critical given their high cost, operational complexity and heterogeneous effects across epidemiological settings. In this study, 64% of communes were estimated to benefit from MDA during the intervention year. While these effects were not sustained beyond that period, ongoing trials of new interventions (for example, malaria vaccines; NCT06578572) may offer fresh opportunities for a longer-lasting impact. Transportability methods could have an important role in identifying optimal areas for deploying these tools.

It is important to note that, in the original trial, the number of MDA rounds administered during the intervention year did not sufficiently cover the entire transmission season, limiting the ability to fully clear the infectious reservoir. Monthly incidence data from the original trial support this notion (Fig. 1), showing a levelling off of effects in November–December 2021, when MDA rounds were stopped. Thus, it is possible that additional MDA rounds during these months would have yielded a stronger intervention impact in both the intervention and postintervention years, thereby potentially increasing the number of communes that could be targeted for MDA.

This study demonstrates how transportability models that leverage publicly available remote sensing datasets can be used to extend trial findings beyond a specific study site to inform intervention targeting. While our study probably did not include all potential effect modifiers, we anticipate that weather, temperature, vegetation indices and population age distribution accounted for substantial heterogeneity in malaria transmission^21,22^, as demonstrated by prior studies^23^. While we explored additional hypothesized modifiers (for example, predicted prevalence of *Plasmodium falciparum* malaria, surface water presence, night-time light radiance and travel time to the nearest health facility), we ultimately excluded these variables due to a lack of overlap with trial sites, insufficient variability or strong collinearity. Thus, we may not have accounted for all potential effect modifiers—a key assumption of transportability models^15^. One key factor our models did not account for (due to a lack of available data) was healthcare system capacity, which is probably a key operational determinant of MDA coverage and effectiveness in real-world settings^24^. However, our validation study demonstrated that the transported estimates for the area surrounding the trial site were highly similar to the original trial estimates, lending credibility to our findings. In future applications, transportability models for malaria interventions could be further strengthened by integrating additional context-specific variables, such as population mobility patterns^25,26^ and local vector species distributions and densities. Moreover, the use of pooled datasets from multiple trials of the same intervention could provide greater variability across epidemiological settings, enabling more robust transportability analyses of malaria intervention effects over large spatial extents^27^.

This study also had several limitations. First, effect modifier data were aggregated to the month level, which may have masked important daily variations for certain variables such as temperature. Second, we did not explicitly account for coverage of other malaria co-interventions (that is, vector control and/or diagnostics, case management, and surveillance capacity), which are also likely modifiers of MDA impact. Thus, our transported MDA estimates implicitly assume that these co-interventions would be implemented before MDA (as recommended by the WHO^8^) and at a coverage similar to that of the trial. Third, given that the number of study clusters was relatively small (*n* = 60), we leveraged temporal variability within the follow-up period to fit transportability models. As such, the number of units with combinations of effect modifier values may have been limited in certain strata. Transportability analyses would theoretically be more robust for trials with a larger number of units and a broader geographical footprint. Fourth, our transported estimates assume that MDA would be delivered similarly in both trial and non-trial areas and are intended to reflect intention-to-treat effects (that is, the predicted effectiveness if non-trial areas had been assigned to receive MDA). Because coverage varied across trial clusters, these estimates also rely on the assumption that coverage patterns in non-trial settings resemble those observed in the trial. Our sensitivity analyses suggest that some of this variation was captured by the measured effect modifiers (for example, precipitation and daytime temperature; Supplementary Appendix 12). However, as coverage can also depend on other factors that are often difficult to predict (for example, community acceptability and sensitization), our estimates may not fully account for this variation. It is also possible that coverage may be lower in real-world settings. Fifth, transportability models were unable to account for preintervention differences in malaria incidence, as in the original trial analysis. In a sensitivity analysis of the original trial, conducted without accounting for preintervention incidence (Supplementary Appendix 9), our point estimates were slightly closer to the null in the intervention year and substantially attenuated towards the null in the postintervention year. Thus, the transported effects for the postintervention year results should be interpreted with caution, as they may represent potentially more conservative, attenuated effects. Sixth, transportability analyses relied on several assumptions, including that all effect measure modifiers were measured and that the models were correctly specified (Methods). Sensitivity analyses were conducted where possible; however, violations of these assumptions cannot be ruled out. Finally, we were able to conduct transportability analyses only in a subset of communes where SMC is implemented in Senegal; other communes were excluded due to limited overlap in effect modifiers with the trial clusters.

## Conclusion

In summary, our study demonstrates the potential of transportability methods as data-driven tools to guide subnational targeting of interventions. Consistent with existing literature, we estimate that, when implemented with high vector control coverage, prompt case management and a robust surveillance system, MDA may yield substantial short-term reductions in malaria burden across broad geographical contexts; however, the effects are probably not sustained beyond the intervention period. In today’s malaria prevention landscape, multiple new and highly effective—but costly—interventions are available (for example, next-generation insecticide-treated nets and malaria vaccines), but funding limitations and other factors make universal roll-out infeasible. Our general approach provides a potential additional framework to support local decision-making on subnational intervention tailoring in this changing landscape.

## Methods

### Analysis overview

We analysed data from a cluster randomized trial (NCT04864444) evaluating MDA for accelerating malaria elimination in low to moderate transmission settings in Senegal. We summarize key trial information below; additional trial details were previously published^4^.

### Study site

The trial was conducted in the Tambacounda Health District of southeastern Senegal, where malaria transmission is low to moderate and highly seasonal, with most cases occurring from July to December. At this site, the National Malaria Programme (NMP) implements standard malaria control interventions, including mass distribution of insecticide-treated nets, health facility-based malaria case management and SMC for children aged 3–120 months. In remote areas with limited healthcare access, Senegal uses malaria case management through the Prise en Charge à Domicile (PECADOM) model, whereby village-level community health workers (dispensateurs de soins à domicile (DSDOMs)) are trained to test and treat febrile malaria cases using rapid diagnostic tests (RDTs) and first-line antimalarials. Some villages use PECADOM+, in which DSDOMs conduct weekly proactive household visits to improve early detection and treatment.

In other regions, malaria endemicity is heterogeneous, driven largely by geography, climate, population density and age distribution. To accommodate varying transmission intensities, the NMP adopts a tailored set of interventions. In the northern and central regions, where transmission is very low due to the semi-arid Sahelian climate, the NMP prioritizes pre-elimination activities consisting of case investigations and proactive response measures. In the central and southern regions, where transmission ranges from low to high due to denser vegetation and higher rainfall, the NMP prioritizes malaria control interventions, including routine net distribution, prompt case management and SMC in highly seasonal areas.

### Study design

The original study was a two-arm, open-label, cluster randomized controlled trial. Sixty villages were randomly selected based on the following eligibility criteria: (1) population size between 200 and 800; (2) location within a health facility catchment area with an annual incidence of 60–160 cases per 1,000 population; and (3) the PECADOM+ model was established or eligible for roll-out. To minimize intervention contamination, a buffer zone of 2.5 km was maintained between village centroids.

### Ethical approval

The study protocol was approved by the Comité National d’Ethique pour la Recherche en Santé (Dakar, Senegal) and the University of California, San Francisco Human Research Protection Program (San Francisco, CA, USA). Stanford investigators had access only to de-identified, cluster-aggregated monthly data.

### Randomization and blinding

Villages were randomized 1:1 using a stratified, constrained randomization approach based on the following covariates: baseline DSDOM presence, health facility, distance to the nearest health facility, baseline microscopy-confirmed malaria prevalence, population in 2019, and the population of children <10 years old in 2019. Participants, investigators and the study team were aware of the randomized assignment, whereas outcome assessors and laboratory technicians were blinded.

### Interventions

During the intervention year (2021), intervention villages received three rounds of MDA with dihydroartemisinin–piperaquine plus a single low dose of primaquine every 6 weeks. Control villages received three rounds of SMC with sulfadoxine–pyrimethamine plus amodiaquine administered to children aged 3–120 months every 4 weeks, according to the standard of care. MDA was administered every 6 weeks based on discussions with the Tambacounda District Medical Office to ensure adequate spacing of drug courses, minimizing potential side effects. The schedule was supported by modelling evidence^7^ suggesting that this interval would provide adequate protection, given piperaquine’s long half-life^28^. The timing also ensured comparable coverage of the transmission season relative to SMC. After the three rounds of MDA, the study villages were followed for an additional year (2022), during which all villages resumed receiving SMC (Fig. 1).

### Procedures

Community sensitization was conducted before the intervention delivery. Before the intervention implementation, all study villages received a mass distribution of pyrethroid–PBO bed nets and year-round PECADOM+. A baseline survey was conducted to assess pyrethroid–PBO net coverage and malaria prevalence (Fig. 1). SMC and MDA were delivered door-to-door using an age-based dosing strategy. All three doses of SMC and MDA were directly observed. During the campaign, suspected cases of malaria were confirmed by RDTs and treated with artemether–lumefantrine. In positive cases, chemoprevention was deferred until the next cycle.

Throughout the study period, data on RDT-confirmed malaria cases were collected from health facilities and PECADOM+ registries. Average village population size was estimated by calculating the mean of the two censuses conducted before and after the intervention implementation. Additional details of the procedures are provided in Supplementary Appendix 13.

### Outcomes

The primary outcome of the trial was village-level *P.falciparum* incidence during the postintervention transmission season (July–December 2022), defined as the number of RDT-confirmed, symptomatic malaria cases divided by the mean village population size (Fig. 1). Malaria incidence during the transmission season of the intervention year was a secondary outcome.

### Effect measure modifiers

We obtained high-resolution data for Senegal on the following potential effect measure modifiers: *P. falciparum* malaria prevalence, precipitation, temperature, vegetation density, percentage of the population under 10 years old and travel time to the nearest health facility. These variables were hypothesized to influence intervention effectiveness by affecting transmission intensity, vector ecology and access to care. For example, moderate rainfall can increase vector breeding sites, while prolonged and heavy rainfall may flush mosquito larvae from breeding sites, thereby reducing malaria transmission^29^. Vegetation density, captured by EVI, may enhance mosquito survival and breeding^30,31^. Temperature has been shown to have a non-linear association with the *Anopheles* biting rate, vector competence, survival and *P. falciparum* development^32^. Population density has a non-monotonic relationship with malaria; transmission models have linked higher population densities to persistent malaria transmission outside of the peak season^33^. The proportion of children is a predictor of transmission intensity, given their disproportionate malaria burden^34^. We ultimately excluded surface water, predicted malaria incidence, predicted malaria prevalence, night-time light radiance, travel time to the nearest health facility and population density from the transportability analyses due to a lack of variation or other reasons detailed in Supplementary Appendix 14.

For estimates of the percentage of the population aged <10 years, we used study data for trial sites and WorldPop data for non-trial sites^35^. Daily precipitation data at 0.05° resolution were sourced from the Climate Hazards Group Infrared Precipitation with Stations (CHIRPS) dataset^36^. Using Google Earth Engine, we aggregated daily data into monthly totals. Mean monthly temperature data at 0.05° resolution were obtained from the MODIS Monthly CMG Land Surface Temperature and Emissivity (MOD21C3)^37^. Because our intent was to transport estimates to small administrative areas, we used a dataset with higher spatial resolution (0.05°) but with monthly, rather than daily, data. Thus, we included only the mean monthly temperature.

While prior studies have shown that daily temperature variation may influence malaria transmission^38^, high-resolution daily remote sensing datasets exhibited missingness rates of 10–20% during high-rainfall months, probably due to cloud cover. Monthly data on the presence of surface water at 30-m resolution were sourced from the Joint Research Centre Monthly Water History, v1.4 dataset^39^. Monthly EVI data at 1-km resolution were obtained from the MODIS/Terra Vegetation Indices Monthly L3 Global 1 km SIN Grid^40^. We extracted the mean values of the percentage of the population under 10 years old and the monthly temperature for the geocoordinates of each trial village centroid and for all communes in Senegal. For precipitation, we calculated the monthly minimum, mean and maximum levels. To account for delayed effects of environmental factors on malaria incidence, we used 1–2-month lags for precipitation, 0–2-month lags for temperature and 0–3-month lags for EVI, informed by prior studies. Justifications for these lags are provided in Supplementary Appendix 14.

### Statistical analysis

First, we assessed the effect modification of the MDA intervention by geographical, environmental and demographic variables. Analyses were conducted at the village-month level across the preintervention, intervention and postintervention transmission seasons (that is, July–December of 2020, 2021 and 2022). Indicator variables were used to test for effect modification by commune. For continuous modifiers, we generated indicators of whether values were above or below the median within each follow-up year. To compare incidence rate ratios for MDA versus control within different levels of potential modifiers, we fitted mixed-effects Poisson regression models restricted to each level of the potential effect modifier, allowing for potential interactions between modifiers and baseline covariates. Models included village-level random intercepts, a log link, an offset for the mean village population size during follow-up and robust standard errors. Consistent with the original trial’s intention-to-treat analysis^4^, malaria case counts were modelled as the dependent variable, with covariates including the following: trial-year fixed effects; an MDA indicator equal to 1 for randomization to MDA in the intervention year (2021) and 0 otherwise; a post-MDA indicator equal to 1 for randomization to MDA in the postintervention year (2022) and 0 otherwise; an indicator equal to 1 for periods and villages with an existing PECADOM+ model and 0 otherwise to account for differential capture of malaria cases at baseline; and variables included in the constrained randomization (health facility, distance to a health facility, baseline microscopy-confirmed malaria prevalence, village population size and population size under 10 years old).

To test for effect modification on the ratio and additive scales^19^, we fitted models with two-way interaction terms between each modifier and year, as well as for the MDA indicator in intervention year analyses and the post-MDA indicator in postintervention year analyses. We defined *R_xz_* as the incidence rate under treatment *x* and modifier *z*. The ratio scale measure of interaction was defined as (*R*_11_*R*_00_)/(*R*_10_*R*_01_), which can be expressed in terms of incidence ratios as IR_11_/(IR_10_IR_01_) (ref. 19). We assessed the additive scale interaction using the relative excess risk due to interaction (RERI), defined as IR_11_ − IR_10_ − IR_01_ + 1 (ref. 19). Because MDA had a protective effect on malaria incidence, we recoded the reference levels for the MDA indicator variable and each modifier to the lowest risk level^41^. We computed standard errors and CIs using the delta method. Effect measure modification analyses were conducted using Stata version 16.

To transport trial estimates to non-trial areas, we applied a doubly robust transportability modelling approach from Dahabreh et al.^20^. We used a non-nested design in which the trial data were concatenated with data from an external target population that partially overlapped with the trial population^42^. When the difference between the trial and external target populations can be characterized by baseline covariates, transportability is conceptually similar to direct standardization across multiple covariates. These methods rely on measuring variables that both modify the intervention effect and differ in distribution between trial and target populations^16^. A flow diagram illustrating the step-by-step procedure of our transportability analyses is provided in Fig. 4.

We restricted analyses to communes where SMC was routinely offered and population density was similar to that of trial sites (≤152 people per 100 m^2^). This was done to minimize potential violations of positivity—that is, to ensure that trial populations could reasonably represent non-trial areas^15^. We combined trial and non-trial data into a single dataset: trial observations included treatment assignment, outcome data and effect modifiers, while non-trial observations included only effect modifiers. An indicator variable for trial participation (*S_i_*) was included in the combined dataset for each village i (for trial data) and commune i (for non-trial data), where *S_i_* = 1 for trial observations and *S_i_* = 0 for non-trial communes. Estimates were transported to the commune level, the smallest standard administrative unit used for public health planning in Senegal. We aggregated malaria incidence data by summing the total number of cases and the population size, and we aggregated effect modifier data by calculating the minimum, mean, maximum or total of covariates as described in the prior section.

The doubly robust approach fits a model for the probability of trial participation and a model for the expectation of the outcome. If either is correctly specified, the estimator is consistent^20^. First, for each commune, we fitted a model for the probability of trial participation Pr(*S_i_* = 1∣*X_i,t_*), where $X_{i,t}$ is a matrix of effect measure modifiers for commune i at month t that includes the variables listed in the previous section. Models were fitted using data from trial sites combined with data from non-trial communes. We then calculated weights by intervention arm ($A_{i}$) in each commune i at month t normalized to the sum of the number of non-trial areas as follows:

(1) $${\hat{w}}_{ait}(X_{it},S_{i},A_{i})=\frac{1-p(X_{it},\hat{\beta })}{p(X_{it};\hat{\beta })e_{a}(X_{it};\hat{\gamma })}\times I(S_{i}=1,A_{i}=a)$$

where $A_{i}$ is an indicator for random assignment to MDA versus control, $p(X_{i,t};\hat{\beta })$ an estimator of the probability that a commune would be included in the trial at month $t\;(\text{Pr}(S_{i}=1|X_{i,t})$, $e_{a}(X_{i,t};\hat{\gamma })$ is an estimator of the probability of assignment to treatment arm a among trial clusters $\left(\text{Pr}(A=a|X_{i,t},S_{i}=1)\right)$, and $I(S_{i}=1|A_{i}=a)$ represents an indicator function equal to 1 for communes that were included in the trial and assigned to arm a and 0 otherwise.

Second, using trial data, we estimated separate outcome models within each intervention arm, modelling monthly malaria cases as a function of effect modifiers. $g_{a}(X_{i,t};\hat{\theta })$ is an estimator for the conditional expectation of the number of malaria cases $\left(E(Y_{i,t}|X_{i,t},S_{i}=1,A_{i}=a)\right)$, where $Y_{i,t}$ is the number of malaria cases for each cluster i at month t. These models were then applied to covariate patterns in non-trial communes to generate arm-specific counterfactual predictions. Outcome models did not adjust for covariates used in the constrained randomization or include a log population offset, as in the original trial^4^, because these data were unavailable in non-trial areas. However, trial estimates were similar with or without these terms (Supplementary Appendix 10). Additionally, we did not adjust for baseline malaria incidence as was done in the original trial due to the lack of such data in non-trial areas. A re-analysis comparing the original trial’s estimates restricted to the intervention year (2021) or the postintervention year (2022) found minimal differences in point estimates and 95% CIs for the intervention year and moderate differences for the postintervention year (Supplementary Appendix 9). However, the interpretation of findings was consistent between the two models, with overlapping 95% CIs.

Given the relatively small number of communes and the potentially large number of covariates, we fitted both trial participation and outcome models using elastic net models with ∝ = 0.5, which use an equal mix of LASSO (least absolute shrinkage and selection operator) and ridge regularization to balance feature selection and the handling of correlated predictors. We fitted models for $g_{a}(X_{i,t};\hat{\theta })$ using a quasi-Poisson family and $p(X_{i,t};\hat{\beta })$ and $e_{a}(X_{i,t};\hat{\gamma })$ using a binomial family. The relax parameter was set to true so that models were refitted without penalty after the initial variable selection, potentially improving prediction accuracy. To identify the optimal level of regularization, we used tenfold cross-validation and a lambda sequence ranging from 0.01 to 100, with a length of 100. We used elastic net model fits with the lambda value that minimized the cross-validated error. If only one covariate was included in the model, we used a generalized linear model with a binomial family instead. Transportability analyses were performed using R version 4.1.0; elastic net models were fitted with the glmnet package (version 4.1.2).

Before fitting the elastic net models, we performed several forms of covariate screening to mitigate multicollinearity, streamline the covariate set and improve model stability. First, we excluded covariates with no overlap between *S* = 1 and *S* = 0 to minimize empirical violations of the positivity of trial participation assumption. Second, we screened covariates for multicollinearity within predefined variable groups (precipitation, temperature and EVI) across lag specifications. For each group of covariates, we calculated pairwise Pearson correlations between variables and estimated their correlations with the outcome. If any correlations were >0.7 within a group of covariates, we retained the covariate with the strongest correlation to the outcome. Third, we screened covariates for inclusion in outcome models using Poisson likelihood ratio tests, retaining those with a *P* value of <0.2. We performed a complete case analysis. Model fit diagnostics are provided in Supplementary Appendix 15.

We then implemented a doubly robust estimator to estimate the intervention effects for each commune. The estimator uses data from trial clusters as well as from non-trial communes. For trial clusters, it uses outcome model predictions as bias correction terms, along with observed outcomes, weighted by the inverse probability of selection into the trial. For non-trial communes in the target population, it relies solely on outcome model predictions. The double robust estimator $\hat{\mu }(a)$ is defined as follows:

(2) $${\hat{\mu }}_{a}={\left\{\sum_{i=1}^{n}{\hat{w}}_{ait}(X_{it},S_{i},A_{i})\right\}}^{-1}\sum_{i=1}^{n}{\hat{w}}_{ait}(X_{it},S_{i},A_{i})\;\;\{Y_{i}-g_{a}(X_{it};\hat{\theta })\}+{\left\{\sum_{i=1}^{n}(1-S_{i})\right\}}^{-1}\sum_{i=1}^{n}(1-S_{i})g_{a}(X_{it};\hat{\theta })$$

We separately estimated weighted averages for the intervention and control arms and calculated percentage effectiveness as (1 – (incident cases in intervention/incident cases in control)) × 100% to estimate the transported effect. To obtain 95% CIs, we performed non-parametric bootstrapping by resampling communes with replacement, using 1,000 iterations.

To validate our transportability model, we transported trial estimates from the intervention year (2021) and the postintervention year (2022) to the geographical area surrounding the trial villages by creating a convex hull of the trial village centroids (Supplementary Appendix 11). Given that effect modifier values should be highly similar to those in geographical areas approximate to the trial site, we expected this analysis to produce effect estimates close to those of the original trial. We extracted effect modifier values from remote sensing data within the trial site, set *S* = 0, and then refitted the transportability models. We obtained 95% CIs using the bias-corrected and accelerated bootstrap method, as described above.

To make causal inferences from our transported estimates, several assumptions are required (Supplementary Appendix 16). First, we assumed conditional exchangeability between clusters assigned to MDA and those assigned to control. Second, we assumed positivity of intervention assignment, meaning that there was a non-zero probability of intervention assignment across covariate strata. We expect the first two assumptions to hold by randomization. Third, we assumed conditional exchangeability over trial participation, meaning that trial clusters were exchangeable with target communes conditional on covariates and study arm. Fourth, we assumed positivity of trial participation, meaning that there was a non-zero probability of trial participation in any covariate stratum needed to ensure conditional exchangeability. To minimize possible violations of this positivity assumption, we restricted analyses to communes where SMC was offered during the trial period and proceeded with estimation only in communes where $p(X_{i,t};\hat{\beta })$ was ≥0.75. Fifth, we assumed consistency, such that each commune’s potential outcome under an intervention equalled its observed outcome when the intervention was implemented similarly in both trial and target settings. We expect this to hold because the trial was designed as a pragmatic trial with MDA delivered as close to routine programmatic conditions as possible. Consistency also requires no interference between clusters; we expect this assumption to hold based on the ≥2.5-km buffer zone between trial clusters^4^. Sixth, we assumed correct model specification. To mitigate violations, we used a doubly robust estimator, which yields consistent estimates if either the trial participation model or the outcome model is correctly specified.

As a sensitivity analysis, we evaluated correlations between MDA coverage and effect modifiers to assess whether the variables used in the transport model could explain heterogeneity in coverage across trial clusters. We calculated Spearman correlation coefficients between coverage and each effect modifier across intervention clusters.

## Extended Data

## Supplementary Material

Supplementary Appendices 1-16 **Supplementary information** The online version contains supplementary material available at https://doi.org/10.1038/s44360-026-00094-1.

## Data Availability

The data used in this secondary analysis were derived from a multi-country collaborative parent clinical trial and consist of aggregated data provided by the parent study team. Data sharing is governed by the data access policies of the parent trial; therefore, data are not publicly available. The analytical dataset used for this study, along with the corresponding data dictionaries, is available on request. All data access requests that comply with ethical and privacy requirements will be reviewed within 2 weeks of receipt, and approved requests will be granted access to the data. Requests should include a brief description of the study objectives. Any queries regarding data requests should be sent via email to M.R. (rohmi@ohsu.edu).

## Code availability

Replication scripts are available on Zenodo at https://doi.org/10.5281/zenodo.18636852 (ref. 43).
