# Supplementary material for: Mapping the local effectiveness of mass drug administration for malaria using transportability methods
Source: Nat Health. 2026 Mar 24;1(7):697–708. doi: 10.1038/s44360-026-00094-1 (PMC13218659; doi:10.1038/s44360-026-00094-1)
Supplement: Supplementary file 1 — Supplementary Information Supplementary Appendixes 1–16. [file 44360_2026_94_MOESM1_ESM.pdf]

nature health

Supplementary information https://doi.org/10.1038/s44360-026-00094-1

# Mapping the local effectiveness of mass drug administration for malaria using transportability methods

In the format provided by the authors and unedited

# SUPPLEMENTARY APPENDIX

## Mapping the local effectiveness of mass drug administration for malaria with transportability methods

*Michelle E. Roh, Yanwei Tong, Gabriella Barratt Heitmann, Junran Jia, El-hadji Konko Ciré Ba, Jean Louis Ndiaye, Ari Fogelson, Paul Milligan, Amadou Seck, Abdoulaye Diallo, Aminata Colle Lo, Michael Baoicchi, Roly Gosling, Adam Bennett, Michelle S. Hsiang, Jade Benjamin-Chung*

---

Table of Contents

**Appendix 1. Potential effect modifier values by study arm in July-December 2021 3**

**Appendix 2. Percent reduction in malaria incidence for MDA vs. control stratified by potential effect modifiers ........ 4**

**Appendix 3. Heterogeneity of the effect of MDA vs. control in the intervention year (2021) ........ 5**

**Appendix 4. Heterogeneity of the effect of MDA vs. control in the post-intervention year (2022) ........ 6**

**Appendix 5. Temporal variation of time-varying effect modifiers during trial follow-up. ........ 7**

**Appendix 6. Characteristics of communes not included in the transportability analysis. ........ 8**

**Appendix 7. Covariate distribution of effect modifiers of Communes within and outside the trial site included in the transportability analysis. ........ 9**

**Appendix 8. Predicted probabilities of trial participation for each commune included in the transportability analysis. ........ 10**

**Appendix 9. Sensitivity analysis of original trial analysis to inclusion of pre-intervention year in the model ........ 12**

**Appendix 10. Sensitivity analysis of original trial analysis to covariate adjustment and log population offset ........ 12**

**Appendix 11. Geographic area surrounding trial villages used to validate transportability analyses. 13**

**Appendix 12. Correlation between MDA coverage and effect modifiers during the intervention year (2021). 14**

**Appendix 13. Details of original randomized trial procedures 15**

**Appendix 14. Additional details about remote sensing data 17**

**Appendix 15. Model fit and predictive accuracy for the probability of trial selection model 20**

**Appendix 16. Identifiability conditions required for valid estimates from transportability analyses 23**

## Appendix 1. Potential effect modifier values by study arm in July-December 2021

| | MDA (n=30) | Control (n=30) |
|---|---|---|
| **Precipitation (mm)** | | |
| Minimum (1-month lag) | 109 (93) | 109 (93) |
| Minimum (2-month lag) | 109 (93) | 109 (93) |
| Mean (1-month lag) | 120 (91) | 120 (91) |
| Mean (2-month lag) | 125 (89) | 125 (89) |
| Maximum (1-month lag) | 109 (93) | 109 (93) |
| Maximum (2-month lag) | 109 (93) | 109 (93) |
| **Temperature (°C)** | | |
| Daytime (0-month lag) | 38.4 (3.9) | 38.4 (4.2) |
| Daytime (1-month lag) | 38.6 (4.1) | 38.6 (4.3) |
| Daytime (2-month lag) | 38.6 (4.2) | 38.7 (4.5) |
| Nighttime (0-month lag) | 25.2 (2.7) | 25.2 (2.7) |
| Nighttime (1-month lag) | 25.6 (2.1) | 25.5 (2.1) |
| Nighttime (2-month lag) | 25.0 (2.4) | 25.0 (2.4) |
| **Enhanced vegetation index** | | |
| 1-month lag | 3683 (1338) | 3700 (1361) |
| 2-month lag | 3675 (1354) | 3682 (1380) |
| **Other** | | |
| Population density (per 3 arc-seconds) | 36.9 (33.2) | 50.4 (39.5) |
| % of population aged < 10 years | 38.1 (4.4) | 37.2 (5.0) |
| Nighttime light radiance | 0.3 (0.1) | 0.3 (0.1) |
| **Travel time to nearest health care facility (minutes)** | | |
| Walking time | 183.0 (112.0) | 179.5 (111.8) |
| Motorized time | 29.0 (21.6) | 29.7 (22.0) |

Columns report the mean (SD) of each variable.

## Appendix 2. Percent reduction in malaria incidence for MDA vs. control stratified by potential effect modifiers

| Effect modifiers | 2021 | 2022 |
|---|---|---|
| **Commune** | | |
| Koussanar | 30% (-21%, 60%) | -3% (-88%, 43%) |
| Sinthiou Maleme | 63% (-31%, 89%) | 44% (-142%, 87%) |
| Netteboulou | 70% (58%, 79%) | -14% (-62%, 20%) |
| Missirah | 60% (-10%, 86%) | 54% (21%, 73%) |
| **Temperature** | | |
| *Nighttime mean* | | |
| Below median | 59% (18%, 80%) | 17% (-56%, 56%) |
| Above median | 41% (-4%, 67%) | 33% (-24%, 64%) |
| *Daytime mean* | | |
| Below median | 64% (34%, 81%) | 24% (-37%, 58%) |
| Above median | 42% (8%, 63%) | 5% (-57%, 42%) |
| **Precipitation** | | |
| *Minimum* | | |
| Below median | 58% (32%, 74%) | 40% (-1%, 64%) |
| Above median | 65% (35%, 82%) | 18% (-55%, 57%) |
| *Mean* | | |
| Below median | 56% (31%, 72%) | 33% (-8%, 59%) |
| Above median | 60% (21%, 79%) | 11% (-65%, 52%) |
| *Maximum* | | |
| Below median | 58% (32%, 74%) | 40% (-1%, 64%) |
| Above median | 65% (35%, 82%) | 18% (-55%, 57%) |
| **Population density** | | |
| Below median | 26% (-31%, 58%) | -22% (-127%, 34%) |
| Above median | 69% (43%, 83%) | 50% (10%, 72%) |
| **% population < 10 years** | | |
| Below median | 64% (33%, 81%) | 57% (25, 75%) |
| Above median | 39% (-12%, 67%) | -22% (-129%, 34%) |
| **Health facility walk time** | | |
| Below median | 50% (7%, 73%) | 21% (-44%, 57%) |
| Above median | 61% (25%, 79%) | 34% (-27%, 66%) |
| **Health facility motorized time** | | |
| Below median | 50% (10%, 72%) | 25% (-34%, 58%) |
| Above median | 59% (22%, 78%) | 34% (-29%, 66%) |
| **Enhanced vegetation index** | | |
| Below median | 56% (27%, 74%) | 33% (-6%, 58%) |
| Above median | 35% (-18%, 64%) | 3% (-86%, 50%) |
| **Nighttime light radiance** | | |
| Below median | 48% (-15%, 77%) | 53% (4%, 77%) |
| Above median | 55% (-11%, 82%) | 34% (-50%, 71%) |

Data are presented as % reduction; 95% CIs are shown in parentheses.

## Appendix 3. Heterogeneity of the effect of MDA vs. control in the intervention year (2021)

| Potential modifier | Observed joint incidence ratio for MDA and modifier | Expected joint incidence ratio for MDA and modifier | Measure of effect modification on ratio scale | Ratio scale p-value | Measure of effect modification on additive scale (95% CI) | Additive scale p-value |
|---|---|---|---|---|---|---|
| **Commune** | | | | | | |
| Koussanar | 1.88 | 0.36 | 5.29 | 0.105 | -3.29 | 0.057 |
| Sinthiou Maleme | 0.70 | 0.45 | 1.57 | 0.659 | -2.76 | 0.510 |
| Netteboulou | 1.12 | 0.47 | 2.39 | 0.706 | -1.54 | 0.231 |
| Missirah | 0.74 | 0.62 | 1.19 | 0.556 | -0.45 | 0.737 |
| **Temperature** | | | | | | |
| Nighttime mean | 1.05 | 0.48 | 2.21 | 0.820 | -1.45 | 0.107 |
| Daytime mean | 1.34 | 0.45 | 2.98 | 0.310 | -2.19 | 0.072 |
| **Precipitation** | | | | | | |
| Minimum | 1.43 | 0.29 | 4.88 | 0.203 | -0.32 | 0.768 |
| Mean | 1.03 | 0.47 | 2.18 | 0.903 | -1.39 | 0.096 |
| Maximum | 1.43 | 0.29 | 4.88 | 0.203 | -0.32 | 0.768 |
| **Other** | | | | | | |
| % population age <10 years | 2.24 | 0.24 | 9.32 | 0.048 | 1.22 | 0.421 |
| Population density | 0.40 | 0.86 | 0.47 | 0.020 | 1.73 | 0.235 |
| Enhanced vegetation index | 1.34 | 0.34 | 3.95 | 0.216 | -0.42 | 0.626 |
| Nighttime light radius | 1.15 | 0.45 | 2.58 | 0.563 | -1.68 | 0.107 |
| Motorized time to healthcare facility | 0.88 | 0.55 | 1.60 | 0.764 | -0.89 | 0.520 |
| Walk time to healthcare facility | 0.83 | 0.49 | 1.70 | 0.662 | -1.81 | 0.260 |

For communes, incidence ratios compare incidence in the MDA arm in a given commune to that in the control arm in all other communes. Incidence ratios were adjusted for timing of case detection relative to full PECADOM+ scale-up, similar to the original trial analysis. Other effect modifiers were coded as 1 for clusters with monthly values above in 2021 and 0 for values below the median. The observed joint incidence ratio compared the malaria incidence for MDA and modifier level 1 vs. control and modifier level 0. The expected joint incidence ratio = (incidence ratio for MDA and modifier level 0 vs. control and modifier level 0) x (incidence ratio for control and modifier level 1 vs. control and modifier level 0). The measure of effect modification on multiplicative scale is the observed joint incidence ratio divided by the expected joint incidence ratio. The measure of effect modification on additive scale is the relative excess risk due to interaction. P-values were obtained by the delta method.

## Appendix 4. Heterogeneity of the effect of MDA vs. control in the post-intervention year (2022)

| Potential modifier | Observed joint incidence ratio for MDA and modifier | Expected joint incidence ratio for MDA and modifier | Measure of effect modification on ratio scale | Ratio scale p-value | Measure of effect modification on additive scale (95% CI) | Additive scale p-value |
|---|---|---|---|---|---|---|
| **Commune** | | | | | | |
| Koussanar | 1.32 | 1.47 | 0.90 | 0.404 | -0.13 | 0.868 |
| Sinthiou Maleme | 0.75 | 1.68 | 0.45 | 0.603 | -0.07 | 0.953 |
| Netteboulou | 1.98 | 1.55 | 1.28 | 0.013 | 0.46 | 0.497 |
| Missirah | 0.64 | 2.09 | 0.31 | 0.036 | -1.31 | 0.005 |
| **Temperature** | | | | | | |
| Nighttime mean | 0.72 | 2.05 | 0.35 | 0.271 | -1.24 | 0.059 |
| Daytime mean | 0.94 | 1.89 | 0.50 | 0.861 | -0.85 | 0.257 |
| **Precipitation** | | | | | | |
| Minimum | 1.69 | 0.95 | 1.79 | 0.099 | -1.78 | 0.003 |
| Mean | 1.41 | 1.42 | 0.99 | 0.352 | 0.01 | 0.995 |
| Maximum | 1.69 | 0.95 | 1.79 | 0.099 | -1.78 | 0.003 |
| **Other** | | | | | | |
| % population age <10 years | 2.31 | 0.80 | 2.88 | 0.008 | -2.27 | 0.001 |
| Population density | 0.56 | 2.51 | 0.22 | 0.091 | -1.80 | 0.008 |
| Enhanced vegetation index | 1.31 | 1.05 | 1.24 | 0.440 | -1.43 | 0.045 |
| Nighttime lights | 1.50 | 1.33 | 1.13 | 0.226 | 0.18 | 0.775 |
| Motorized time to healthcare facility | 0.97 | 1.76 | 0.55 | 0.939 | -0.71 | 0.286 |
| Walk time to healthcare facility | 0.93 | 1.66 | 0.56 | 0.832 | -0.50 | 0.450 |

For communes, incidence ratios compare incidence in the MDA arm in a given commune to that in the control arm in all other communes. Incidence ratios were adjusted for timing of case detection relative to full PECADOM+ scale-up, similar to the original trial analysis. Other effect modifiers were coded as 1 for clusters with monthly values above in 2022 and 0 for values below the median. The observed joint incidence ratio compared the malaria incidence for MDA and modifier level 1 vs. control and modifier level 0. The expected joint incidence ratio = (incidence ratio for MDA and modifier level 0 vs. control and modifier level 0) x (incidence ratio for control and modifier level 1 vs. control and modifier level 0). The measure of effect modification on multiplicative scale is the observed joint incidence ratio divided by the expected joint incidence ratio. The measure of effect modification on additive scale is the relative excess risk due to interaction. P-values were obtained by the delta method.

## Appendix 5. Temporal variation of time-varying effect modifiers during trial follow-up.

Includes data from communes included in the transportability analysis: N=122 in the intervention year (2021) and N=119 in the post-intervention year (2022). Colored lines indicate means within each commune, and black lines means within the trial clusters.

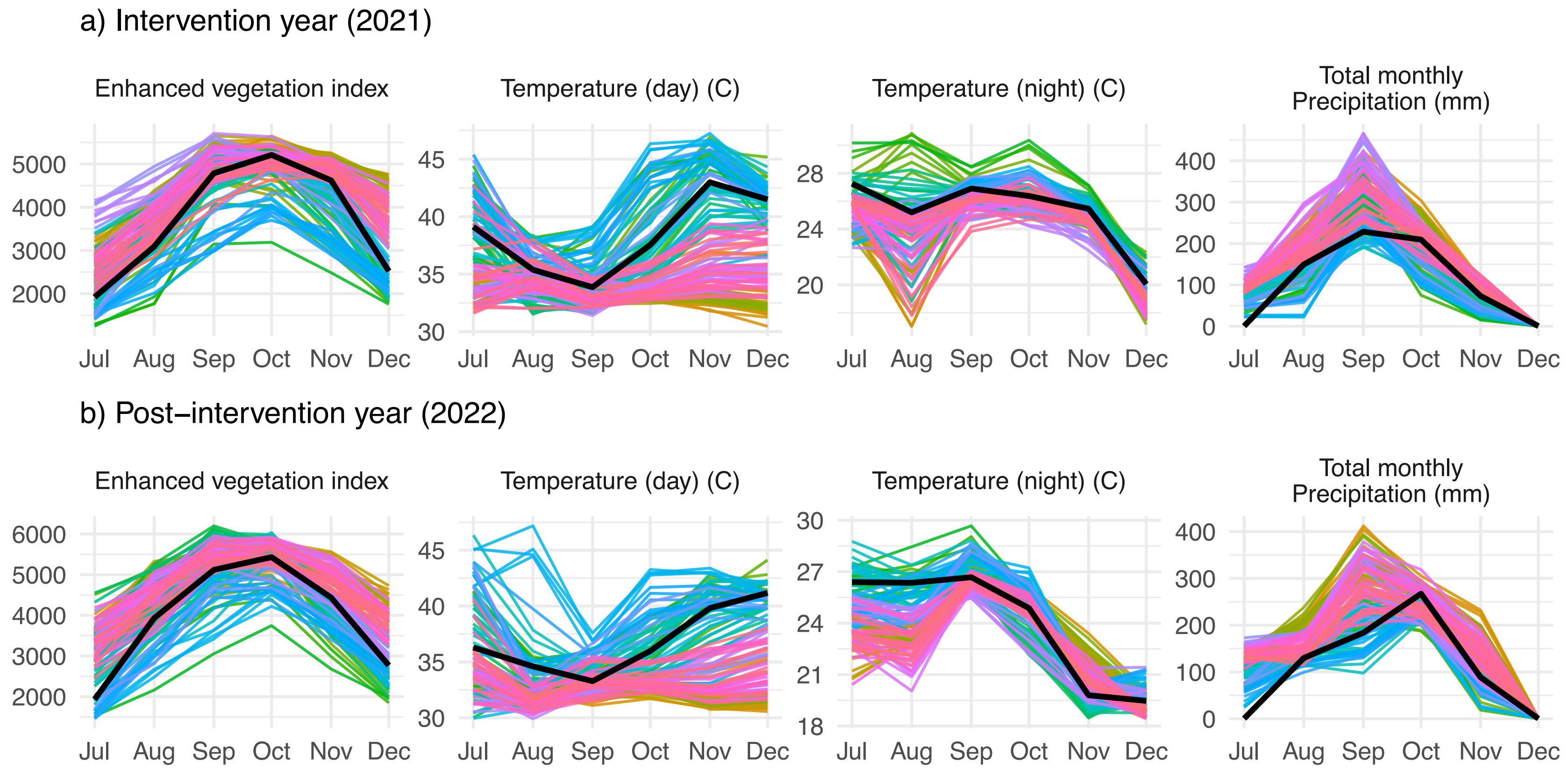

# Appendix 6. Characteristics of communes not included in the transportability analysis.

This subset of communes includes those with a population size >152 per km, with a predicted probability of trial participation ≤0.75, or where SMC was not offered during the trial period. The color of each point indicates the standardized mean difference for a given covariate in a given commune, calculated as the mean in a non-trial commune minus the mean in the trial site divided by the pooled standard deviation. Communes are sorted from north to south.

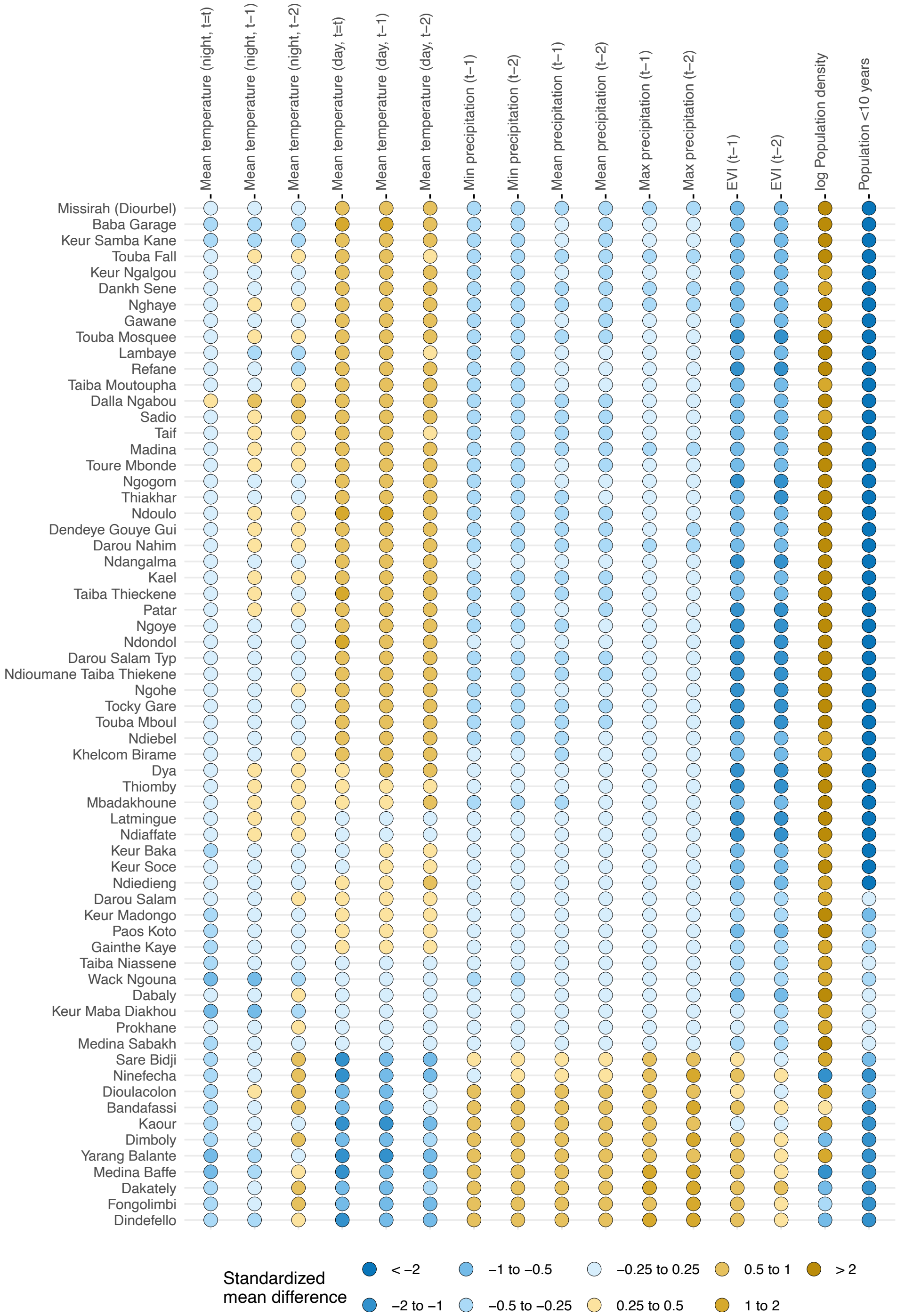

## Appendix 7. Covariate distribution of effect modifiers of Communes within and outside the trial site included in the transportability analysis.

Data for trial villages are shown in orange, and data for Communes included in the transportability analysis are shown in blue. The number of observations is shown as village-months in the trial site and as Commune-months outside the trial site.

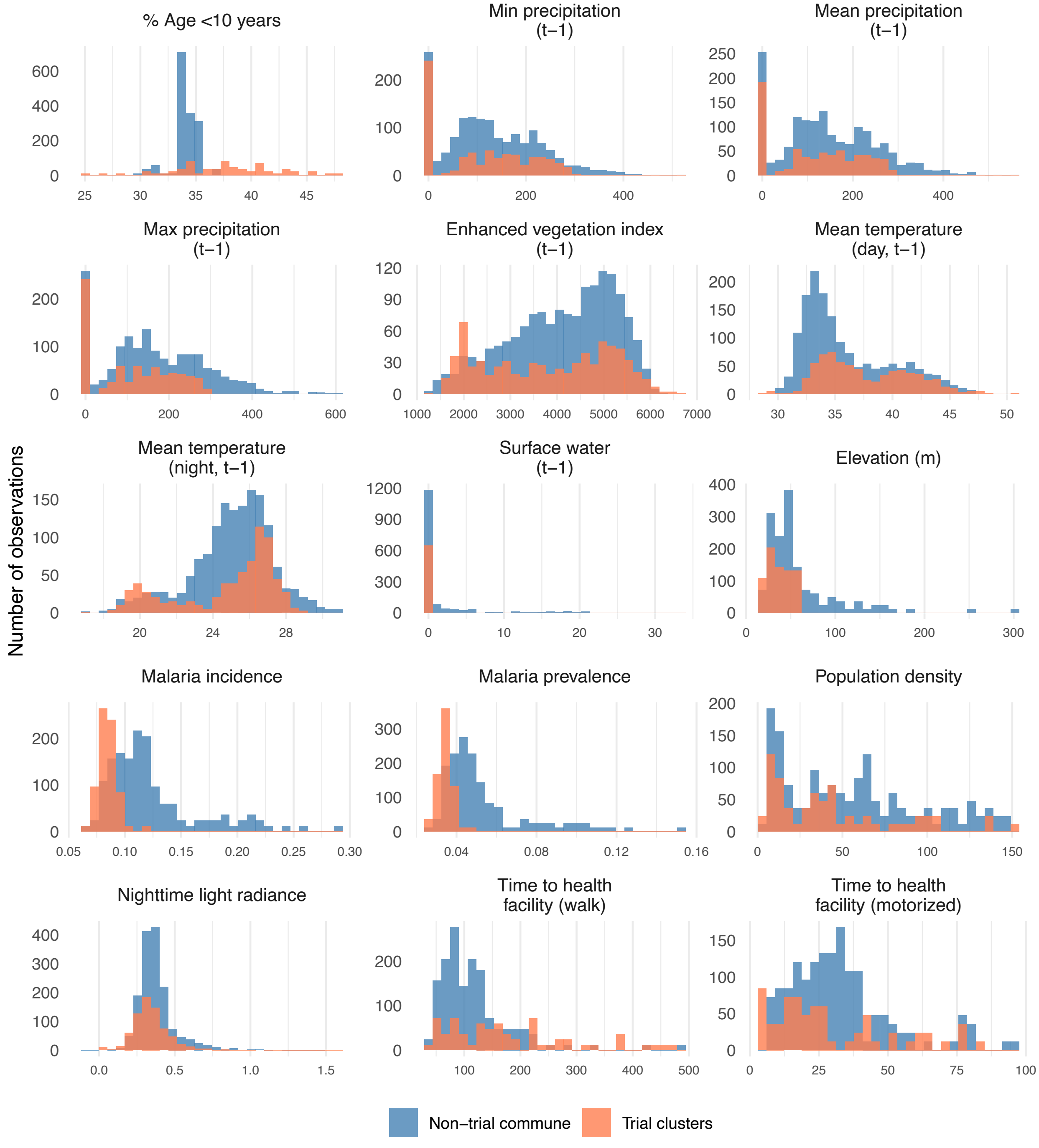

# Appendix 8. Predicted probabilities of trial participation for each commune included in the transportability analysis.

Predicted probabilities P(S) were obtained from elastic net regression models for analyses in which the P(S)>0.75. Analyses were performed separately for each commune using monthly data from July to December (N=366 per commune). Data for trial villages are shown in orange, and data for Communes included in the transportability analysis are shown in blue. Covariates considered for trial participation models included precipitation, temperature, enhanced vegetation index, and population density. The final covariate list used in models varied between communes following screening for collinearity, data sparsity, and feature selection using elastic net regression. Analyses restricted to communes with population size $\leq$152 per km and where SMC was offered during the trial period. Common support = the percentage of months in a given non-trial Commune for which P(S) was within the range of the study clusters' P(S) for that analysis.

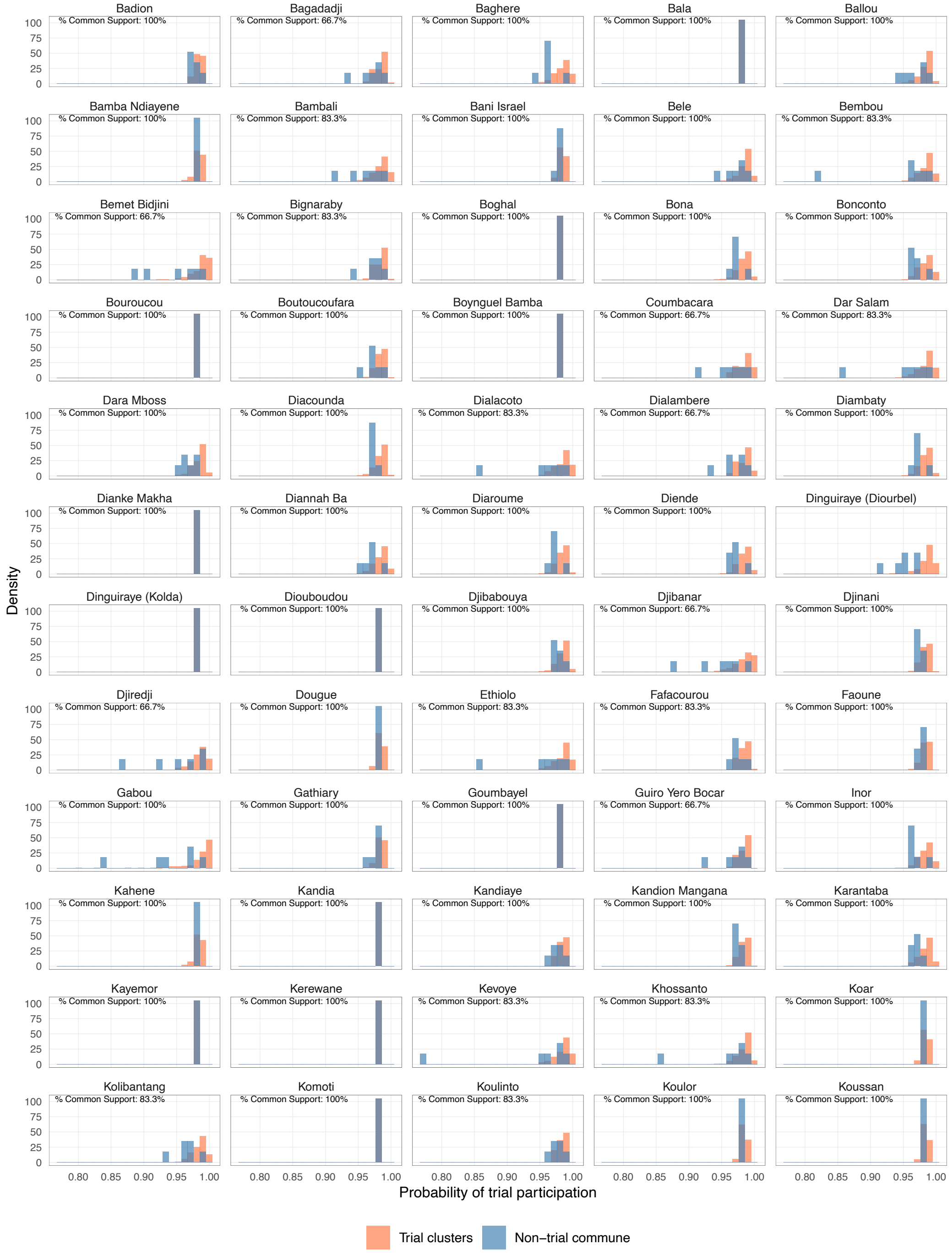

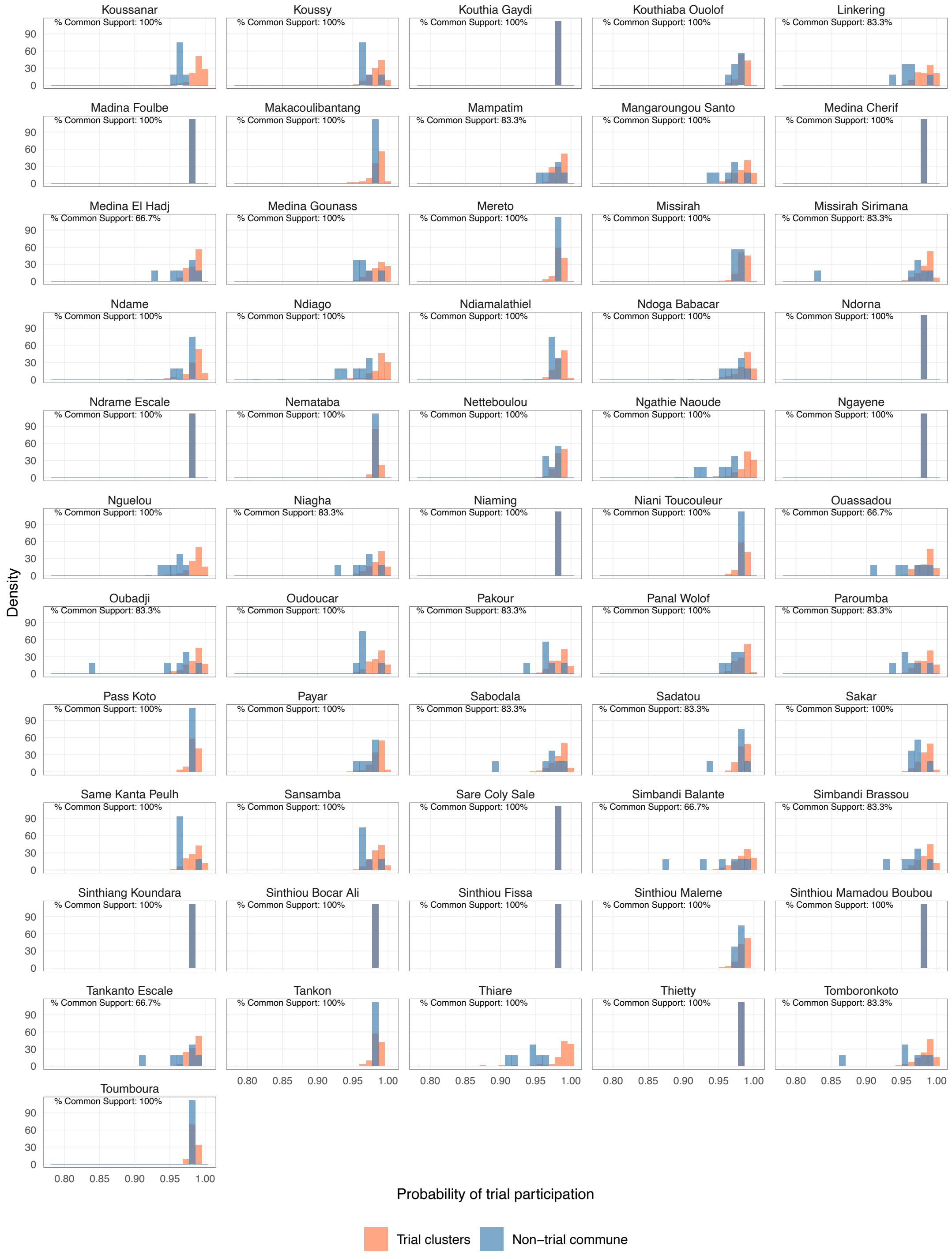
Koussanar
% Common Support: 100%
Koussy
% Common Support: 100%
Kouthia Gaydi
% Common Support: 100%
Kouthiaba Ouolof
% Common Support: 100%
Linkering
% Common Support: 83.3%
Madina Foulbe
% Common Support: 100%
Makacoulibantang
% Common Support: 100%
Mampatim
% Common Support: 83.3%
Mangaroungou Santo
% Common Support: 100%
Medina Cherif
% Common Support: 100%
Medina El Hadj
% Common Support: 66.7%
Medina Gounass
% Common Support: 100%
Mereto
% Common Support: 100%
Missirah
% Common Support: 100%
Missirah Sirimana
% Common Support: 83.3%
Ndame
% Common Support: 100%
Ndiago
% Common Support: 100%
Ndiamalathiel
% Common Support: 100%
Ndoga Babacar
% Common Support: 100%
Ndorna
% Common Support: 100%
Ndrame Escale
% Common Support: 100%
Nemataba
% Common Support: 100%
Netteboulou
% Common Support: 100%
Ngathie Naoude
% Common Support: 100%
Ngayene
% Common Support: 100%
Nguelou
% Common Support: 100%
Niagha
% Common Support: 83.3%
Niaming
% Common Support: 100%
Niani Toucouleur
% Common Support: 100%
Ouassadou
% Common Support: 66.7%
Oubadji
% Common Support: 83.3%
Oudoucar
% Common Support: 100%
Pakour
% Common Support: 83.3%
Panal Wolof
% Common Support: 100%
Paroumba
% Common Support: 83.3%
Pass Koto
% Common Support: 100%
Payar
% Common Support: 100%
Sabodala
% Common Support: 83.3%
Sadatou
% Common Support: 83.3%
Sakar
% Common Support: 100%
Same Kanta Peulh
% Common Support: 100%
Sansamba
% Common Support: 100%
Sare Coly Sale
% Common Support: 100%
Simbandi Balante
% Common Support: 66.7%
Simbandi Brassou
% Common Support: 83.3%
Sinthiang Koundara
% Common Support: 100%
Sinthiou Bocar Ali
% Common Support: 100%
Sinthiou Fissa
% Common Support: 100%
Sinthiou Maleme
% Common Support: 100%
Sinthiou Mamadou Boubou
% Common Support: 100%
Tankanto Escale
% Common Support: 66.7%
Tankon
% Common Support: 100%
Thiare
% Common Support: 100%
Thietty
% Common Support: 100%
Tomboronkoto
% Common Support: 83.3%
Toumboura
% Common Support: 100%
Density
0
30
60
90
0.80
0.85
0.90
0.95
1.00
Probability of trial participation
Trial clusters
Non−trial commune

## Appendix 9. Sensitivity analysis of original trial analysis to inclusion of pre-intervention year in the model

| | Intervention year (2021) | | Post-intervention year (2022) | |
|---|---|---|---|---|
| | **Pre-intervention** | **No pre-intervention** | **Pre-intervention** | **No pre-intervention** |
| Unadjusted | 55% (28%, 71%) | 47% (23%, 63%) | 26% (-18%, 53%) | 15% (-16%, 37%) |
| Adjusted* | 55% (28%, 71%) | 49% (25%, 66%) | 26% (-17%, 53%) | 4% (-28%, 29%) |

Table cells show intervention effectiveness, defined as (1 - IRR for MDA vs. control) x 100%, where IRR is the malaria incidence rate ratio. 95% confidence intervals are shown in parentheses.

* Adjusted for trial-year fixed effects (minus 2020 baseline data for ‘no pre-intervention’ analyses), an indicator variable equal to 1 for periods and villages with an existing PECADOM model and 0 otherwise to account for differential capture of malaria cases at baseline, and variables included in the constrained randomization (i.e., health post of village, distance to health post, baseline microscopy-confirmed malaria prevalence, village population size, and population size of children <10 years)

## Appendix 10. Sensitivity analysis of original trial analysis to covariate adjustment and log population offset

| | Intervention year (2021) | | Post-intervention year (2022) | |
|---|---|---|---|---|
| | **Offset** | **No offset** | **Offset** | **No offset** |
| Unadjusted in original analysis* | 55% (28%, 71%) | 55% (27%, 72%) | 26% (-18%, 53%) | 26% (-20%, 55%) |
| Fully unadjusted | 52% (21%, 71%) | 52% (21%, 71%) | 21% (-20%, 54%) | 21% (-26%, 51%) |
| Adjusted† | 55% (28%, 71%) | 55% (28%, 71%) | 26% (-17%, 53%) | 26% (-18%, 55%) |

Table cells show intervention effectiveness, defined as (1 - IRR for MDA vs. control) x 100%, where IRR is the malaria incidence rate ratio. 95% confidence intervals are shown in parentheses.

* Included an indicator equal to 1 in periods when proactive community case management of fever occurred in the village and 0 otherwise to account for differential capture of malaria cases at baseline.

† Adjusted for trial-year fixed effects, an indicator variable for periods and villages with an existing PECADOM model, and variables included in the constrained randomization (i.e., health post of village, distance to health post, baseline microscopy-confirmed malaria prevalence, village population size, and population size of children <10 years

## Appendix 11. Geographic area surrounding trial villages used to validate transportability analyses.

To validate our transportability model, we transported trial estimates to the area surrounding the trial villages (highlighted in green), approximated by a convex hull of trial village centroids. Because effect modifier values in this area were expected to closely resemble those in the trial villages, transported estimates were anticipated to align with original trial results.

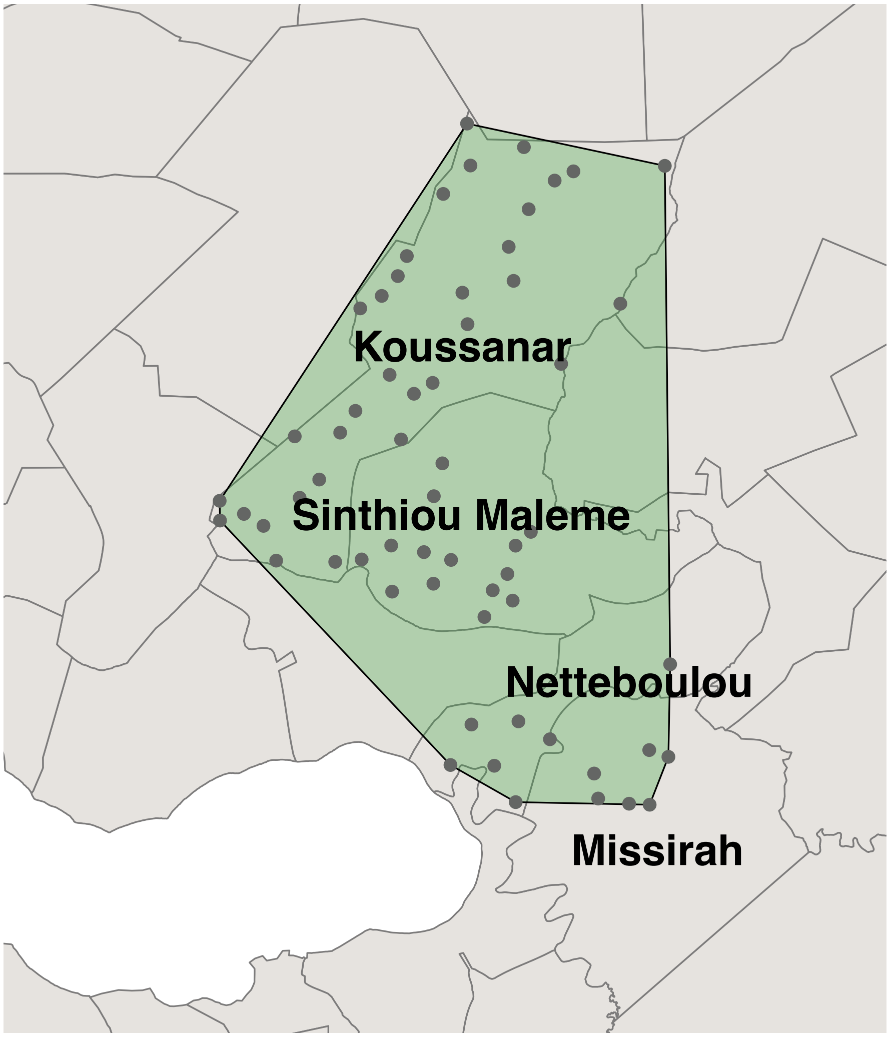

# Appendix 12. Correlation between MDA coverage and effect modifiers during the intervention year (2021).

In the original trial, MDA coverage varied from 54–80% across intervention clusters. Thus, our estimates of transported effects may also be partially explained by differences in coverage between trial clusters in addition to variation in demographic and environmental settings. To evaluate whether some of this effect could be partially explained by the effect modifiers considered for our transportability analyses, we calculated Spearman's correlation coefficients between MDA coverage and each effect modifier. Analyses were conducted only among intervention trial clusters. Y-axis represents the median village-level coverage of all three doses of dihydroartemisinin-piperaquine, averaged across the three rounds. Effect modifier values were calculated by extracting effect modifier values from each village; for time-varying values (e.g., precipitation, temperature, and EVI), we took the mean values across the 2021 transmission season (July-December). As shown in the plots, correlations were generally weak (|ρ| < 0.2) for most modifiers, except for daytime temperature, precipitation, and travel time to nearest health facility.

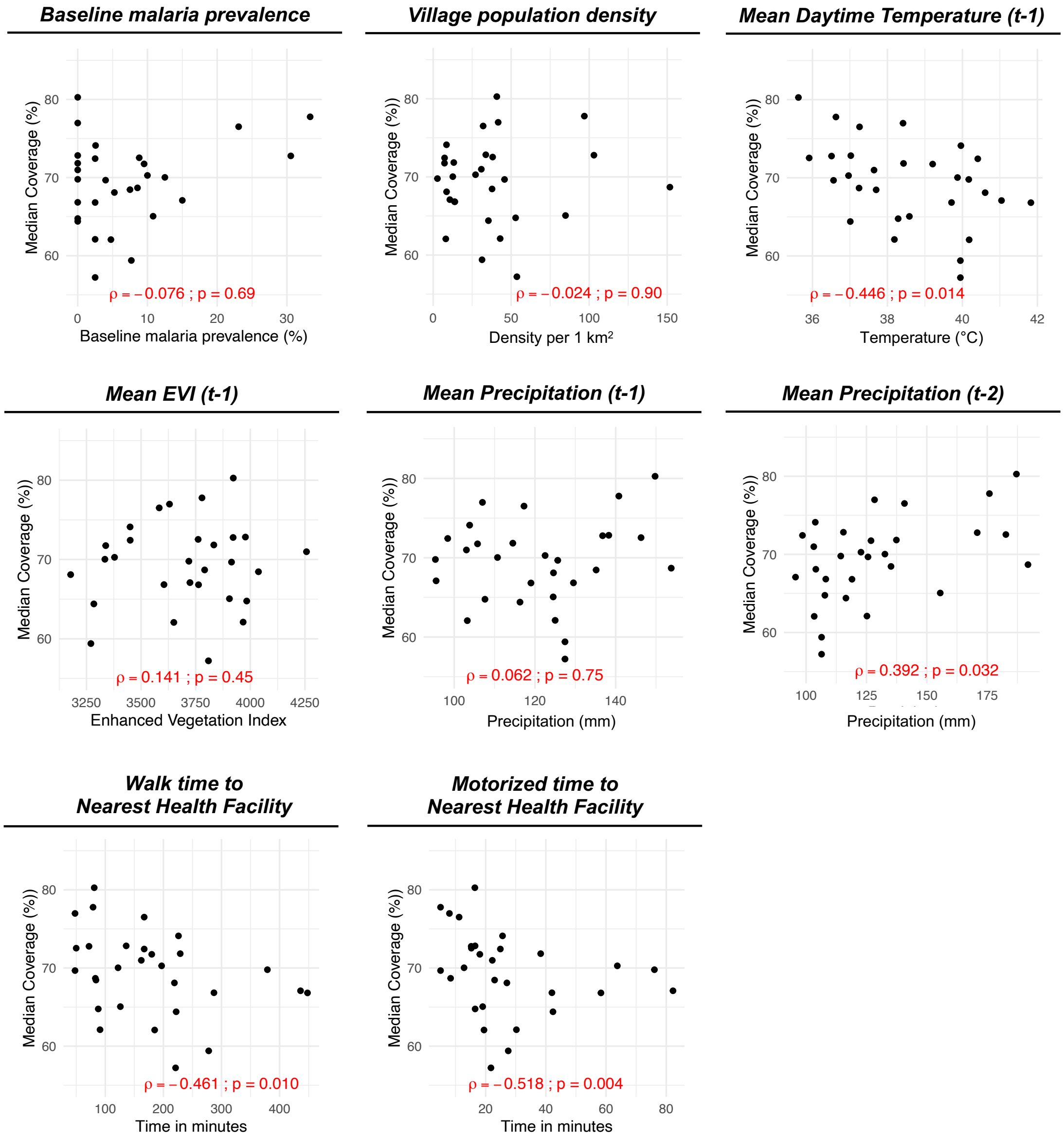

## Appendix 13. Details of original randomized trial procedures

Upon selection of eligible villages, study staff engaged administrative, health, and religious leaders in Tambacounda to present trial objectives, outline activities, and obtain approval. Community sensitization included village visits, town hall meetings, household visits, and media campaigns (radio, television, and social media) prior to campaign launch.

Before intervention implementation, all study villages received mass distribution of pyrethroid-PBO bednets and year-round PECADOM+. A baseline survey was conducted to assess pyrethroid-PBO net coverage and malaria prevalence. At each round, residents aged ≥3 months were screened and enrolled upon informed consent. Exclusion criteria included self-reported severe or chronic illness, hypersensitivity to MDA drugs, pregnancy, recent antimalarial use (within three weeks), or use of medications affecting cardiac function or prolonged QTc (within four weeks). Participants were excluded from single low-dose primaquine if breastfeeding or <2 years old. SMC was administered by the NMP to children aged 3-120 months, excluding those with acute illness or fever, hypersensitivity to SMC drugs, recent antimalarial use within 3 weeks or sulfonamide use within ten days. SMC and MDA were delivered door-to-door using an age-based dosing strategy (**Table 1**). All three doses of SMC and MDA were directly observed. During the campaign, suspected cases of malaria were confirmed by RDT and treated with artemether-lumefantrine. Positive cases deferred chemoprevention until the next cycle.

Data on RDT-confirmed malaria cases were collected using health facility and PECADOM+ registries. Average village population size was estimated by taking the mean across two censuses conducted before and after intervention implementation.

**Table 1. Dosing regimens for dihydroartemisinin-piperaquine, single-low dose primaquine, and sulfadoxine-pyrimethamine + amodiaquine**

**Mass drug administration (intervention arm):**

| **Dihydroartemisinin-piperaquine** | | | |
|---|---|---|---|
| Age | Number of tablets containing 40 mg dihydroartemisinin and 320 mg piperaquine | | |
| | Day 1 | Day 2 | Day 3 |
| 3 to 24 months | 0.5 | 0.5 | 0.5 |
| 2 to 7 years | 1 | 1 | 1 |
| 8 to 10 years | 1.5 | 1.5 | 1.5 |
| 11 to 14 years | 2 | 2 | 2 |
| 15+ years | 3 | 3 | 3 |
| **Primaquine (given on day 1)** | | | |
| Age | Number of tablets | Dosage (mg) | Volume (ml) of water used to dilute tablets |
| 2 to 4 years | 0.5 | 3.75 | 3 |
| 5 to 7 years | 0.75 | 5.625 | 5 |
| 8 to 10 years | 1 | 7.5 | 10 |
| 11 to 13 years | 1.5 | 11.25 | -- |
| 14+ years | 2 | 15 | -- |

A. **Seasonal malaria chemoprevention (control arm):**

| **Sulfadoxine-pyrimethamine + amodiaquine (SP-AQ)** | | | |
|---|---|---|---|
| Age | Number of tablets containing 500 mg sulfadoxine and 25 mg pyrimethamine, and 150 mg amodiaquine | | |
| | Day 1 | Day 2 | Day 3 |
| 3 to 11 months | 0.5 tablet SP + 0·5 tablet AQ | 0.5 tablet AQ | 0.5 tablet AQ |
| 12 to 59 months | 1 tablet SP + 1 tablet AQ | 1 tablet AQ | 1 tablet AQ |
| 60 to 120 months | 1.5 tablets SP + 1.5 tablets AQ | 1.5 tablets AQ | 1.5 tablets AQ |

## Appendix 14. Additional details about remote sensing data

### Details on effect measure modifiers excluded from transportability analyses

Data on 2020 *Pf* prevalence data in children 2–10 years of age was obtained from the Malaria Atlas Project at 5m resolution.[1] Predicted population size per 1 $km^2$ was obtained from WorldPop.[2] Nighttime light radiance (as a proxy for socioeconomic status) were sourced at monthly resolution from the Visible Infrared Imaging Radiometer Suite (VIIRS) Day/Night Band.[3] Walking and motorized time (in minutes) to nearest health facility were extracted from Malaria Atlas Project at 1 km resolution.[4] We extracted the mean values of parasite prevalence, population density, % population under 10 years, monthly temperature, and monthly percentage of any surface water for the geocoordinates of each trial village centroid and for all Communes in Senegal.

Predicted surface water was zero in all trial villages and most non-trial Communes, except in a small number of Communes. The distributions of predicted malaria incidence and prevalence of Malaria Atlas Project estimates[1] were lower in the trial site than in non-trial areas (**Appendix 6**); additionally, MAP estimates had minimal variation across trial clusters and did not correlate with the baseline prevalence in trial clusters (which was assessed by microscopy among all ages), suggesting MAP values did not serve as a strong proxy for overall incidence or prevalence (**Figure 1**). The range of nighttime light radiance within trial sites was small and did not fully overlap with non-trial sites (-0.09 to 1.0 in trial sites versus 0.04 to 4.2 in non-trial sites). We excluded travel time to health facility because model algorithms did not converge when included, resulting in unstable estimates. We excluded population density because it was highly collinear with % of population >10 years, and including age resulted in more stable models.

**References:**

1. Hay, S. I. & Snow, R. W. The Malaria Atlas Project: Developing Global Maps of Malaria Risk. *PLoS Med* **3**, e473 (2006).
2. Tatem, A. J. WorldPop, open data for spatial demography. *Sci Data* **4**, 170004 (2017).
3. Lee, T. E. *et al.* The NPOESS VIIRS Day/Night Visible Sensor. *Bull Amer Meteor Soc* **87**, 191–200 (2006).
4. Weiss, D. J. *et al.* Global maps of travel time to healthcare facilities. *Nat Med* **26**, 1835–1838 (2020).


### Justification of lag periods for environmental effect measure modifiers

The choice of lags were informed by the following studies: a systematic review of *Plasmodium falciparum* seasonality transmission reported that the most commonly found significant lag in the Sahel region for rainfall was 3 months, while 0–1 month lags were found to be significant for temperature.[1] Studies of rainfall and malaria in Senegal found associations at 1–2 month lags.[2] A more recent model of malaria seasonality in Senegal that was tested in other West African countries in the Sahel region found that malaria incidence peaked 1–2 months after the peak of the rainy season for most countries, and incidence peaked 3 months after the rainy season peak in Niger.[3] A systematic review of *Plasmodium falciparum* transmission seasonality globally found that EVI was associated with transmission from 0–3 months.[1]

**References:**

1. Reiner, R. C., Geary, M., Atkinson, P. M., Smith, D. L. & Gething, P. W. Seasonality of Plasmodium falciparum transmission: a systematic review. *Malar J* **14**, 343 (2015).
2. Fall, P., Diouf, I., Deme, A. & Sene, D. Assessment of Climate-Driven Variations in Malaria Transmission in Senegal Using the VECTRI Model. *Atmosphere* **13**, 418 (2022).
3. Diouf, I. *et al.* Climate Variability and Malaria over West Africa. *Am J Trop Med Hyg* **102**, 1037–1047 (2020).

**Figure 1. Comparison of observed baseline malaria prevalence vs. predicted malaria prevalence.**

In the original trial, baseline *Plasmodium falciparum* malaria prevalence was assessed by microscopy in all ages at the end of the transmission season (December 10-20, 2020). Malaria Atlas Project predicts *Plasmodium falciparum* malaria prevalence at fine spatial scales among children 2-10 years using a combination of rapid diagnostic test- and microscopy-based surveys.

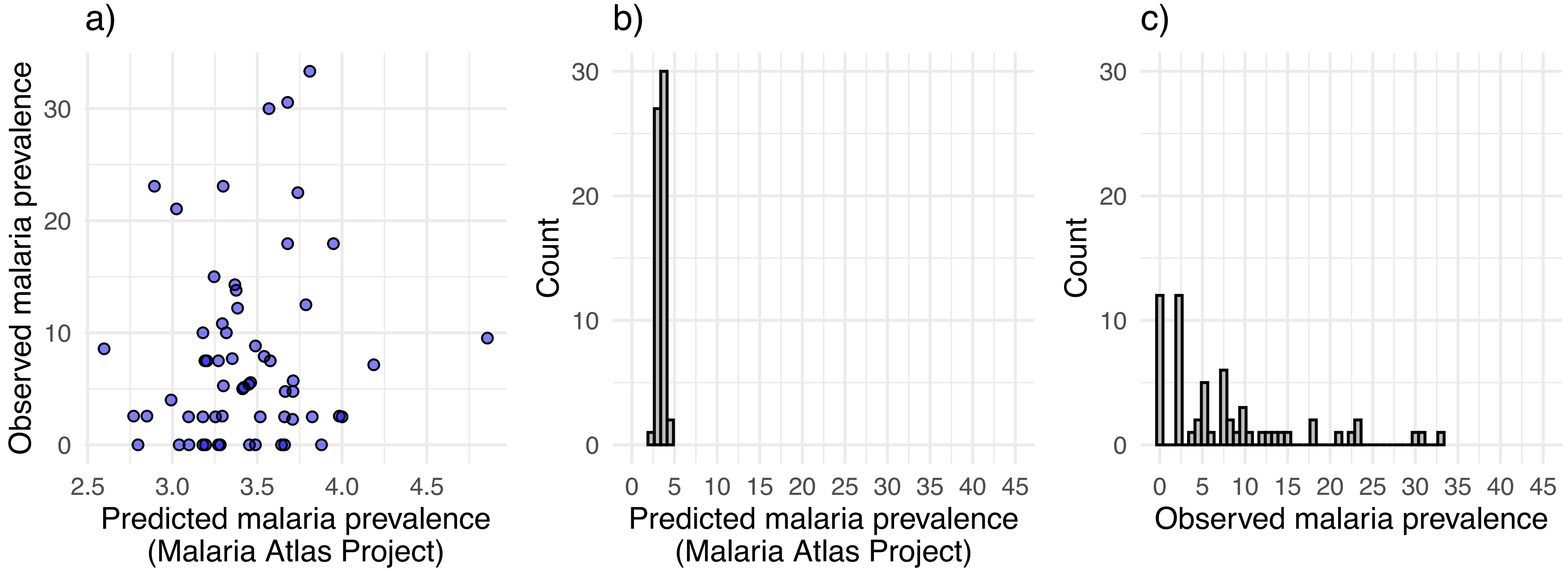

## Appendix 15. Model fit and predictive accuracy for the probability of trial selection model

For our transportability analyses, we used elastic net regression to model the probability of trial participant and for outcome models. Below, we provide model diagnostics for both models.

### Trial participation model diagnostics

The Area Under the Curve (AUC) ranged from 0.5 to 0.96 (mean=0.76) (**Figure 1**). Out of the 129 transportability analyses, the AUC was 0.5 in 25 models (19%) in which no covariates were retained by elastic net (**Figure 2**). Model fit was stronger when a larger number of covariates were retained by the elastic net model. In the other 81% of models, the AUC values were adequate (0.7 ≤ AUC <0.8) in 38 models, good in 61 models (0.8 ≤ AUC <0.9), and excellent in 18 models (AUC ≥ 0.9). AUCs were high in the Communes overlapping with the trial and in the areas with the strongest estimated transported effects (**Figure 3**). All Brier scores were <0.1, indicating excellent model calibration; however, this largely reflects the low prevalence of non-trial units relative to trial units as the dependent variable.

**Figure 1.** Distribution of area under the curve in the models of trial selection (N=129 transportability analyses)

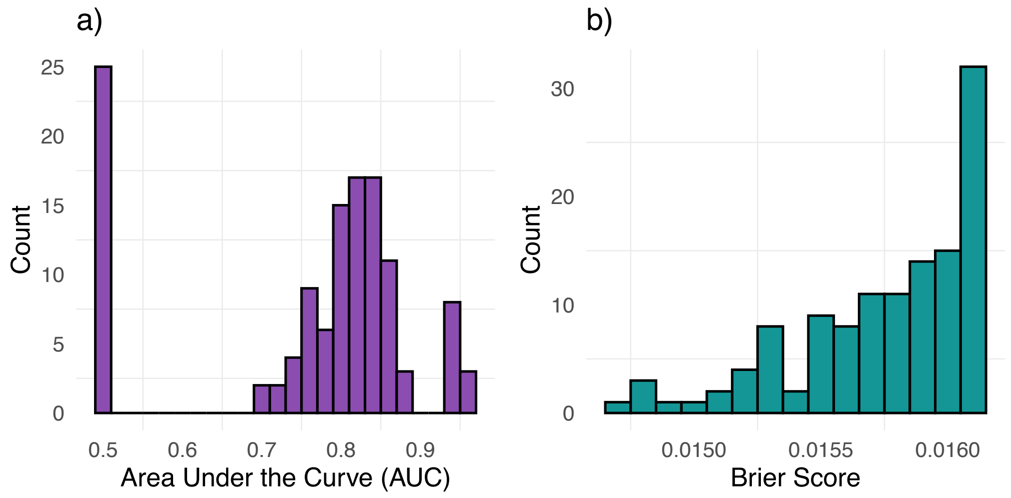

**Figure 2.** Area under the curve in the models of trial selection by the number of covariates retained by the elastic net model

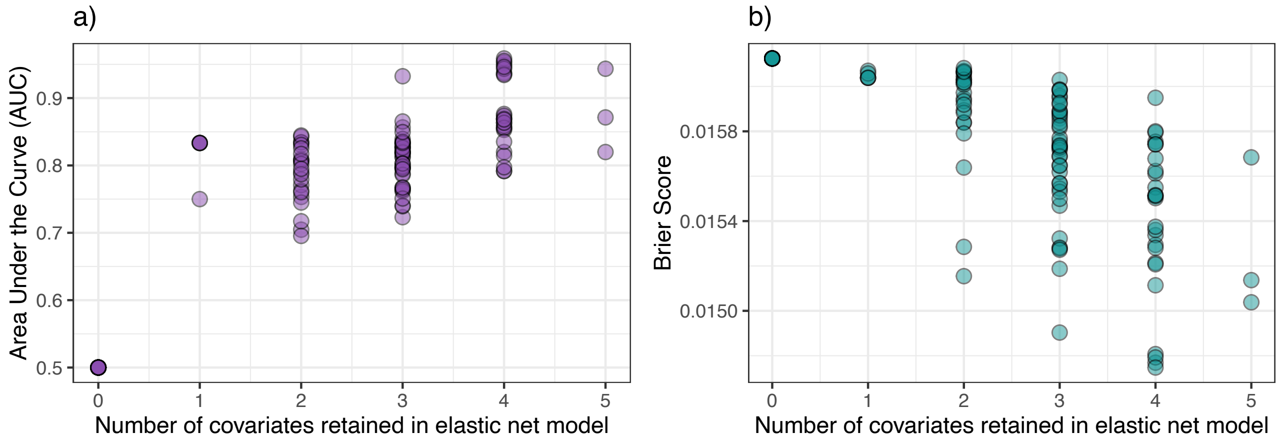


**Figure 3.** Map of Area Under the Curve in the trial selection models

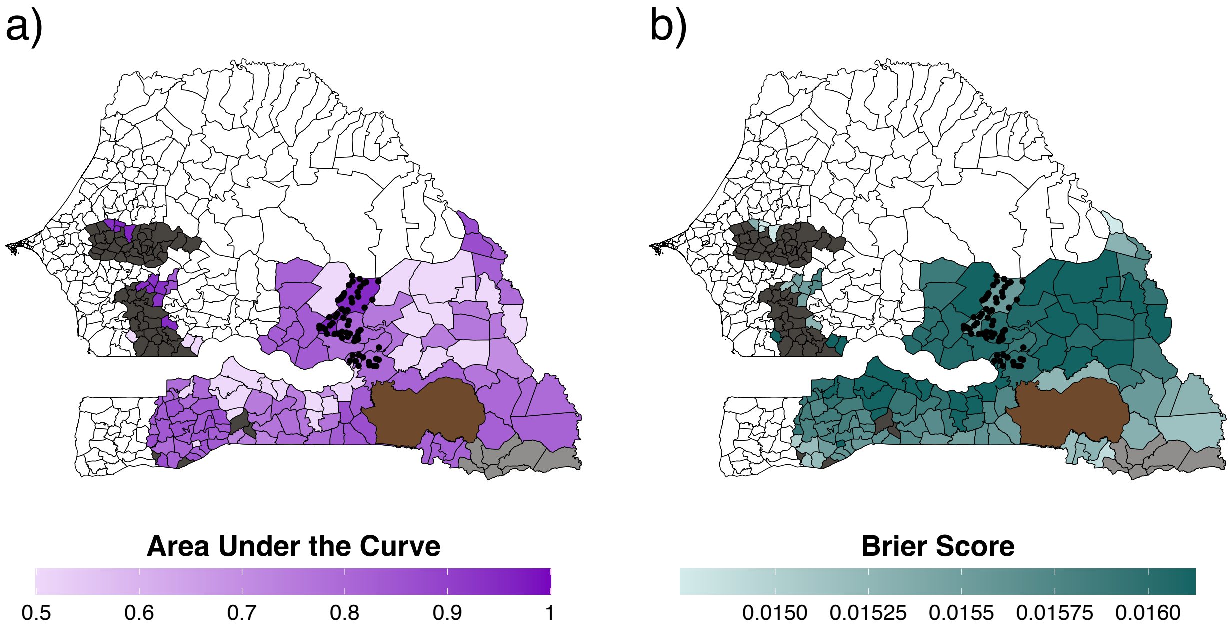

## Outcome model diagnostics

Outcome models demonstrated moderate predictive accuracy with pseudo-$R^2$ values of 0.42 and 0.44 for the treatment and control arms respectively. Mean absolute errors were modest at 4.1 and 7.6 events for the treatment and control arms. Cross-validation identified lambda = 0.01 as the penalty minimizing deviance for both models; the more parsimonious models using the 1-SE rule corresponded to lambda values of 2.0 and 5.1 for the treatment and control arms respectively. Both models showed substantial overdispersion (dispersion parameters of 6.1 and 13.9), as is common in models of infectious disease case counts[1] (Lloyd-Smith et al., 2005).

**Table 1.** Predictive accuracy of the outcome models

| | Mean absolute error | Pseudo-$R^2$ | Lambda minimum value | Lambda 1-SE value | Dispersion |
|---|---|---|---|---|---|
| Treatment arm model | 4.1 | 0.42 | 0.01 | 2.0 | 6.1 |
| Control arm model | 7.6 | 0.44 | 0.01 | 5.1 | 13.9 |

**Figure 4.** Calibration plots for the outcome models

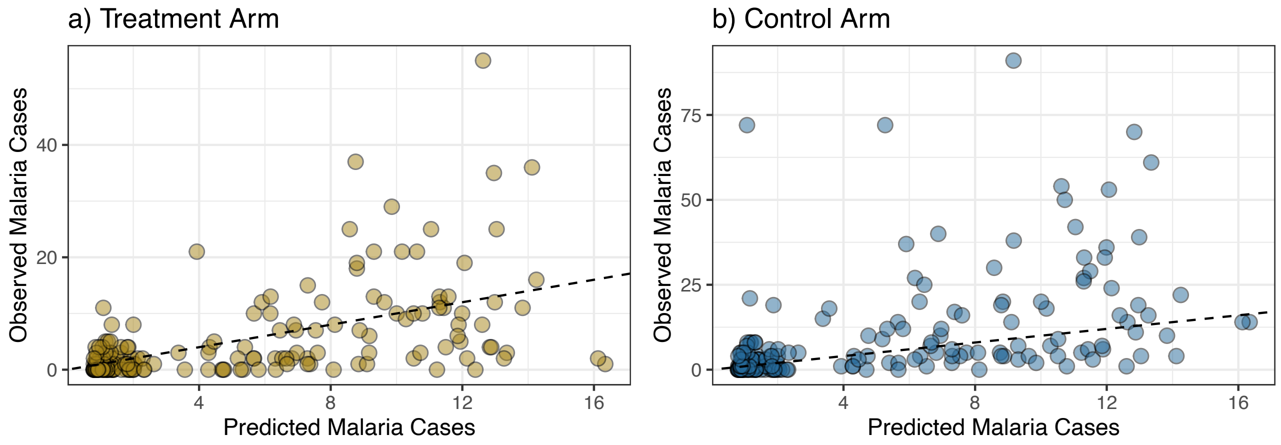



**Reference:**

1. Lloyd-Smith JO, Schreiber SJ, Kopp PE, Getz WM. Superspreading and the effect of individual variation on disease emergence. *Nature* 2005; 438: 355–9.

## Appendix 16. Identifiability conditions required for valid estimates from transportability analyses

The following table summarizes the key assumptions required for identifying and transporting causal effects from a trial to a target population as described by Dahabreh et al (2020).[1] Before presenting the assumptions, we define the key variables used throughout:

- $A$ = intervention assignment
- $Y$ = outcome; $Y^a$ = potential outcome of each unit/cluster under intervention assignment $a$
- $X$ = vector of covariates/effect modifiers used for transportability analyses
- $S$ = binary indicator of cluster selection into the trial; $s^*$denotes enrollment in the original trial

| # | Assumptions |
|---|---|
| 1 | **Conditional exchangeability of intervention assignment in the trial.**<br>For each treatment $a \in A$ and each $x$ with $f(x, S = s^*) > 0$,<br>$$E[\,Y^a\,|X = x, S = s^*, Z = z] = E[\,Y^a\,|X = x, S = s^*]$$<br>States that intervention assignment is independent of a cluster's potential outcomes (i.e., no unmeasured confounding). Assumption 1 is expected to hold by intervention randomization. |
| 2 | **Positivity of intervention assignment in the trial.**<br>For each treatment $a \in A$,<br>$$0 < P[A = a\,|X = x, S = s^*] < 1$$<br>States that every cluster has a non-zero probability of being assigned to intervention, ensuring that for every combination of covariates in the population, there are both treated and untreated clusters in the trial. Assumption 2 is expected to hold by intervention randomization. |
| 3 | **Conditional exchangeability in measure between the trial and target population.**<br>For each pair of treatments $a$ and $a'$in $A$ and for every $x$ with $f(x, S = 0) > 0$,<br>$$E\left[Y^a - Y^{a'}\,\middle|\,X = x, S = 0\right] = E\left[Y^a - Y^{a'}\,\middle|\,X = x, S = s^*\right]$$<br>States that all effect measure modifiers that differ between the trial and target population are measured and correctly included in transportability analyses. While not directly testable, sensitivity analyses showed that transportability models produced similar estimates to the original trial when applied to the areas immediately surrounding trial villages (**Appendix 11**). |
| 4 | **Positivity of the probability of trial participation.**<br>$$P[S = s^*\,|\,X = x] > 0 \text{ for all } x \text{ with } f(x, S = 0) > 0$$<br>States that all covariates required to transport effects occur with a non-zero probability in trial and target populations. To mitigate risks in violating Assumption 4, non-trial Communes where SMC was not offered as standard-of-care during the trial period and where the probability of trial participation was <0.75 were excluded. The |

| | |
|---|---|
| | distribution of covariates and probabilities of trial participation between trial and non-trial Communes are shown in **Appendices 7-8**. |
| 5 | **Consistency assumption.**<br>If $A_i = a$, then $Y_i^a = Y_i$ for every unit $i$ in trial $s^*$or the target population.<br>States that the observed outcome equals the cluster's observed outcome under assigned intervention. This assumes that the intervention is implemented similarly in both trial and non-trial settings and that there is no interference between clusters (i.e., a cluster's potential outcome is unaffected by intervention assignment of other clusters). We expect this assumption to hold because (1) the study was designed as a pragmatic trial and (2) a ≥2.5 km buffer zone was maintained between clusters. |
| 6 | **Correct model specification.**<br>States that models estimating trial participation probabilities and outcomes must correctly specified. For transportability analyses that use doubly robust estimators, only one of the two models need to be correctly specified. To reduce the risk of model misspecification, we used a doubly robust estimator and elastic net regression with covariates selected via 10-fold cross-validation to flexibly model a large number of covariates. Sensitivity analyses were also conducted to validate our transportability models by transporting estimates to areas immediately surrounding trial villages. |

**References:**

1. Dahabreh IJ, Petito LC, Robertson SE, Hernán MA, Steingrimsson JA. Toward Causally Interpretable Meta-analysis: Transporting Inferences from Multiple Randomized Trials to a New Target Population. *Epidemiol.* 2020 May;31(3):334-344. doi: 10.1097/EDE.0000000000001177. PMID: 32141921; PMCID: PMC9066547.
