# Supplementary material for: Mapping the local effectiveness of mass drug administration for malaria using transportability methods
Source: Nat Health. 2026 Mar 24;1(7):697–708. doi: 10.1038/s44360-026-00094-1 (PMC13218659; doi:10.1038/s44360-026-00094-1)
Supplement: Supplementary file 2 — Reporting Summary [file 44360_2026_94_MOESM2_ESM.pdf]

Corresponding author(s): Jade Benjamin-Chung

Last updated by author(s): Feb 10, 2026

Reporting Summary

Nature Portfolio wishes to improve the reproducibility of the work that we publish. This form provides structure for consistency and transparency in reporting. For further information on Nature Portfolio policies, see our Editorial Policies and the Editorial Policy Checklist.

Statistics

For all statistical analyses, confirm that the following items are present in the figure legend, table legend, main text, or Methods section.

n/a	Confirmed	
☐	☒	The exact sample size (*n*) for each experimental group/condition, given as a discrete number and unit of measurement
☐	☒	A statement on whether measurements were taken from distinct samples or whether the same sample was measured repeatedly
☐	☒	The statistical test(s) used AND whether they are one- or two-sided<br>*Only common tests should be described solely by name; describe more complex techniques in the Methods section.*
☐	☒	A description of all covariates tested
☐	☒	A description of any assumptions or corrections, such as tests of normality and adjustment for multiple comparisons
☐	☒	A full description of the statistical parameters including central tendency (e.g. means) or other basic estimates (e.g. regression coefficient) AND variation (e.g. standard deviation) or associated estimates of uncertainty (e.g. confidence intervals)
☐	☒	For null hypothesis testing, the test statistic (e.g. *F*, *t*, *r*) with confidence intervals, effect sizes, degrees of freedom and *P* value noted<br>*Give P values as exact values whenever suitable.*
☒	☐	For Bayesian analysis, information on the choice of priors and Markov chain Monte Carlo settings
☐	☒	For hierarchical and complex designs, identification of the appropriate level for tests and full reporting of outcomes
☐	☒	Estimates of effect sizes (e.g. Cohen's *d*, Pearson's *r*), indicating how they were calculated

Our web collection on statistics for biologists contains articles on many of the points above.

Software and code

Policy information about availability of computer code

Data collection	N/A
Data analysis	Remote sensing data was retrieved using Google Earth Engine JavaScript code. Statistical analyses were performed in Stata and R. Specifically, effect measure modification analyses were conducted in Stata, version 16. Transportability analyses were performed using R version 4.1.0; elastic net models were fit with the glmnet package (version 4.1.2). Replication scripts are available at https://doi.org/10.5281/zenodo.18636852.

For manuscripts utilizing custom algorithms or software that are central to the research but not yet described in published literature, software must be made available to editors and reviewers. We strongly encourage code deposition in a community repository (e.g. GitHub). See the Nature Portfolio guidelines for submitting code & software for further information.

Data

Policy information about availability of data

All manuscripts must include a data availability statement. This statement should provide the following information, where applicable:

- Accession codes, unique identifiers, or web links for publicly available datasets
- A description of any restrictions on data availability
- For clinical datasets or third party data, please ensure that the statement adheres to our policy

The data used in this secondary analysis were derived from a multi-country collaborative parent clinical trial and consist of aggregated data provided by the parent

study team. Data sharing is governed by the data access policies of the parent trial and is therefore not publicly available. The analytic dataset used for this study and/or the de-identified individual participant data and corresponding data dictionaries may be available upon request and approval by EKCB and MSH. Data access requests will be reviewed and a response provided within two weeks of receipt. Requests should include a brief description of study objectives. Any queries for data requests should be made via email to Michelle Roh (email: rohmi@ohsu.edu).

Research involving human participants, their data, or biological material

Policy information about studies with human participants or human data. See also policy information about sex, gender (identity/presentation), and sexual orientation and race, ethnicity and racism.

Reporting on sex and gender	This study analyzed cluster-level outcomes from a cluster-randomized trial. We did not perform gender or sex-stratified analyses because there was not evidence of effect heterogeneity by these variables in the original trial's analysis.
Reporting on race, ethnicity, or other socially relevant groupings	Analyses did not account for race/ethnicity. The study population is from Senegal and is of African ancestry.
Population characteristics	See below
Recruitment	Not applicable. This is an analysis of an existing dataset.
Ethics oversight	Ethical approval was provided by the Comité National d'Ethique pour la Recherche en Santé (Dakar, Senegal) and the University of California, San Francisco Human Research Protection Program (San Francisco, CA, USA). The US Centers for Disease Control and Prevention and Population Services International agreed to rely on the Institutional Review Board of the University of California, San Francisco for ethical oversight of the trial.

Note that full information on the approval of the study protocol must also be provided in the manuscript.

Field-specific reporting

Please select the one below that is the best fit for your research. If you are not sure, read the appropriate sections before making your selection.

☐ Life sciences ☒ Behavioural & social sciences ☐ Ecological, evolutionary & environmental sciences

For a reference copy of the document with all sections, see nature.com/documents/nr-reporting-summary-flat.pdf

Behavioural & social sciences study design

All studies must disclose on these points even when the disclosure is negative.

Study description	This study was a secondary analysis of quantitative data from a cluster-randomized trial of mass drug administration for malaria conducted in Tambacounda district, Senegal from September 2020 to December 2022.
Research sample	The analysis used data from a cluster-randomized trial of mass drug administration for malaria. The study population included all individuals in study clusters and were representative of the study sample, although the purpose of transportability analyses was to generate generalizable findings to the larger Senegal population. The trial pouplation, where the study took place, included individuals who resided in Tambacounda district, Senegal, a low-to-moderate malaria transmission setting. As the analyses used aggregated data, no direct information is available on demographics, except from extrapolated values, which reported approximately 38% of children <10 years of age resided in the population.
Sampling strategy	We included data from all individuals recorded in the baseline survey and subsequent incident malaria cases detected through surveillance.
Data collection	No new data was collected for this study. The manuscript Methods section includes a brief summary of data collection in the original trial.
Timing	September 2020 to December 2022
Data exclusions	All cluster-level data from the original trial were included in this analysis.
Non-participation	Not applicable.
Randomization	Villages were randomized at a 1:1 ratio using a stratified, constrained randomization approach of the following covariates: baseline presence of DSDOMs, health facility, distance to nearest health facility, baseline microscopy-confirmed malaria prevalence, population in 2019, and village population of children <10 years in 2019. An independent trial investigator generated 50 000 randomization schemes and one was randomly sampled from the top 1% (n=500) with the lowest balance scores. Participants, investigators, and the study team were aware of randomized assignment; outcome assessors and laboratory technicians were blinded.

Reporting for specific materials, systems and methods

We require information from authors about some types of materials, experimental systems and methods used in many studies. Here, indicate whether each material, system or method listed is relevant to your study. If you are not sure if a list item applies to your research, read the appropriate section before selecting a response.

Materials & experimental systems

n/a	Involved in the study
☒	☐ Antibodies
☒	☐ Eukaryotic cell lines
☒	☐ Palaeontology and archaeology
☒	☐ Animals and other organisms
☐	☒ Clinical data
☒	☐ Dual use research of concern
☒	☐ Plants

Methods

n/a	Involved in the study
☒	☐ ChIP-seq
☒	☐ Flow cytometry
☒	☐ MRI-based neuroimaging

Clinical data

Policy information about clinical studies

All manuscripts should comply with the ICMJE guidelines for publication of clinical research and a completed CONSORT checklist must be included with all submissions.

Clinical trial registration	NCT04864444
Study protocol	The original trial protocol is included in the appendix of the primary outcomes manuscript from the trial: https://doi.org/10.1016/S1473-3099(24)00741-2
Data collection	No new data was collected for this study. The manuscript Methods section includes a brief summary of data collection in the original trial.
Outcomes	The primary outcome of the trial was village-level, Plasmodium falciparum-confirmed malaria incidence during post-intervention transmission season (July–December 2022), defined as the number of RDT-confirmed, symptomatic malaria cases divided by the mean village population size calculated based on two censuses conducted pre- and post-MDA. Malaria incidence during the transmission season of the intervention year was considered a secondary outcome. Both outcomes were pre-defined in the study protocol of the main manuscript.

Plants

Seed stocks	*Report on the source of all seed stocks or other plant material used. If applicable, state the seed stock centre and catalogue number. If plant specimens were collected from the field, describe the collection location, date and sampling procedures.*
Novel plant genotypes	*Describe the methods by which all novel plant genotypes were produced. This includes those generated by transgenic approaches, gene editing, chemical/radiation-based mutagenesis and hybridization. For transgenic lines, describe the transformation method, the number of independent lines analyzed and the generation upon which experiments were performed. For gene-edited lines, describe the editor used, the endogenous sequence targeted for editing, the targeting guide RNA sequence (if applicable) and how the editor was applied.*
Authentication	*Describe any authentication procedures for each seed stock used or novel genotype generated. Describe any experiments used to assess the effect of a mutation and, where applicable, how potential secondary effects (e.g. second site T-DNA insertions, mosiacism, off-target gene editing) were examined.*
