# Supplementary material for: Mapping the local effectiveness of mass drug administration for malaria using transportability methods
Source: Nat Health. 2026 Mar 24;1(7):697–708. doi: 10.1038/s44360-026-00094-1 (PMC13218659; doi:10.1038/s44360-026-00094-1)
Supplement: Supplementary file 3 — Peer Review File [file 44360_2026_94_MOESM3_ESM.pdf]

nature portfolio

Peer Review File

Mapping the local effectiveness of mass drug administration for malaria with transportability methods

Corresponding Author: Dr Jade Benjamin-Chung

This file contains all reviewer reports in order by version, followed by all author rebuttals in order by version.

Version 0:

Reviewer comments:

Reviewer #1

(Remarks to the Author)
In this study, the authors applied transportability analysis to extrapolate the results from a recent cluster-randomized trial to non-trial areas in Senegal. Overall, the analysis is well conducted, and the manuscript is clearly written. I believe this paper provides an excellent applied example of transportability analysis, an area where empirical demonstrations remain limited compared with the number of theoretical contributions. I only have a few minor comments:

1. As the authors briefly mention, one of the important and challenging assumptions of transportability analysis is conditional exchangeability across study samples. Although I agree that this application adds valuable evidence for local decision-making, it is also important to highlight that the transported effect does not necessarily reflect the causal effect in the target population unless all assumptions, including S-admissibility and correct model specification, are satisfied. The conclusion may need to be a little bit toned down.

2. While the authors used a doubly robust estimator, this does not ensure correct model specification. It would be fair to clarify this point in the limitations section.

3. I am not familiar with the specific context in Senegal, but can we be confident about the consistency assumption? In other words, could MDA implementation differ between trial and non-trial areas? If so, please clarify this point.

4. Some graphical presentation might help readers understand the concept of transportability analysis.

(Remarks on code availability)

Reviewer #2

(Remarks to the Author)

The paper describes models to predict the impact of MDA in populations living in the same region but their villages were not included in the trial. This is of value because MDAs can help in malaria control but are resource intensive. Being able to predict whether such an expensive intervention will be promising is today more important than ever before as funding of global health intervention is shrinking precipitously.

One of the most important variables predicting high impact of antimalarial MDAs is coverage. A highly transmissible organism like P. falciparum requires high coverage to make a lasting impact. Coverage is difficult to predict. Qualitative scientists have produced considerable amounts of research and still predictions are tricky. Within trials considerable variability has been documented between randomized villages. The authors do acknowledge the problem but have not included the unpredictability of coverage as a major limitation of their predictions.

The authors write “travel time to health facility may serve as a proxy for MDA coverage in real-world implementation settings, and …”. Travel time may play a role but it is far from a correlate. The reasons why people participate or do not participate in

MDAs are complex. Reducing this complexity to travel time is an oversimplification. I understand the absence of alternatives but the authors should acknowledge this limitation.

Minor point. In Figure 3 the authors use the label ‘c)’ for two panels. Clearly one should be c) and the next d)…

(Remarks on code availability)

Reviewer #3

(Remarks to the Author)
A very well-written and methodologically rigorous manuscript that presents a novel application of doubly robust transportability methods to a cluster-randomized malaria trial in Senegal. The integration of ecological and demographic effect modifiers is commendable and contributes meaningfully to the literature on subnational targeting of malaria interventions, adding both operational relevance and policy utility.
To enhance transparency and interpretability, I recommend the following improvements: (1) Include model fit diagnostics to support the robustness of transported estimates. While the modeling framework is clearly described, the manuscript does not report predictive accuracy metrics (e.g., RMSE, calibration plots). A brief summary or supplemental figure would strengthen confidence in the outcome models.
(2) The analytic pipeline spans multiple modeling steps but lacks a visual or tabular summary. A schematic or table outlining the workflow and model structure would improve reproducibility and help readers navigate the complexity.
(3) Although the causal assumptions underlying transportability are discussed, a concise summary table would make them easier to reference and evaluate, particularly for readers less familiar with causal inference frameworks.
Addressing these points would improve clarity and accessibility, and further elevate the manuscript’s contribution to evidence-based malaria control.

(Remarks on code availability)

Open Access This Peer Review File is licensed under a Creative Commons Attribution 4.0 International License, which permits use, sharing, adaptation, distribution and reproduction in any medium or format, as long as you give appropriate credit to the original author(s) and the source, provide a link to the Creative Commons license, and indicate if changes were made.
In cases where reviewers are anonymous, credit should be given to 'Anonymous Referee' and the source.
The images or other third party material in this Peer Review File are included in the article’s Creative Commons license, unless indicated otherwise in a credit line to the material. If material is not included in the article’s Creative Commons license and your intended use is not permitted by statutory regulation or exceeds the permitted use, you will need to obtain permission directly from the copyright holder.
To view a copy of this license, visit https://creativecommons.org/licenses/by/4.0/

Response to Peer Review Comments

Please find below our responses to reviewers' comments. Each comment is enumerated followed by our responses, referencing the **page and line numbers** of the cleaned version where revisions were made.

Reviewer #1:

Overall response: In this study, the authors applied transportability analysis to extrapolate the results from a recent cluster-randomized trial to non-trial areas in Senegal. Overall, the analysis is well conducted, and the manuscript is clearly written. I believe this paper provides an excellent applied example of transportability analysis, an area where empirical demonstrations remain limited compared with the number of theoretical contributions. I only have a few minor comments:

1. As the authors briefly mention, one of the important and challenging assumptions of transportability analysis is conditional exchangeability across study samples. Although I agree that this application adds valuable evidence for local decision-making, it is also important to highlight that the transported effect does not necessarily reflect the causal effect in the target population unless all assumptions, including S-admissibility and correct model specification, are satisfied. The conclusion may need to be a little bit toned down.

Response: We appreciate the reviewer's comment and have made changes to the Conclusions paragraph to tone down our conclusions and in the Limitations paragraph to note that violations to these assumptions cannot be ruled out.

- **Conclusions paragraph (Page 8, lines 337-346):**

"In summary, our study demonstrates the ~~value of~~ potential for transportability methods as a data-driven tool to guide subnational targeting of interventions. Consistent with existing literature, we estimate that, when implemented with high vector control coverage, prompt case management, and a robust surveillance system, MDA ~~can~~ may yield significant short-term reductions in malaria burden across broad geographic contexts, though effects are likely not sustained beyond the intervention period. In today's malaria prevention landscape, multiple new and highly effective but costly interventions are available (e.g., next-generation insecticide treated nets and malaria vaccines), but funding limitations and other factors make universal rollout infeasible. Our general approach provides a ~~rigorous evidence base~~ framework to support local decision-making on subnational intervention tailoring in this changing landscape."

- **Discussion section (Page 8, lines 329-332):**

"Sixth, transportability analyses relied on several assumptions, including that all effect measure modifiers were measured and that models were correctly specified (see

Methods). Sensitivity analyses were conducted where possible, but violations of these assumptions cannot be ruled out."

2. While the authors used a doubly robust estimator, this does not ensure correct model specification. It would be fair to clarify this point in the limitations section.

<u>Response:</u> We thank the reviewer for this comment and agree that correct model specification is needed for a causal interpretation, even with a double robust estimator. To clarify this point in the manuscript, we updated our Methods section to explicitly list correct model specification as an important assumption and revised the limitations paragraph of the Discussion section to include a broader statement regarding the reliance of our analyses on these assumptions. Please see below for the following changes:

- **Methods section (Page 15, lines 663-666):**

"[To make causal inferences about intervention effectiveness outside the original trial areas, several assumptions are required]… Sixth, transportability analyses require correct model specification. To account for potential violations of this assumption, we used a doubly robust estimator, which yields consistent estimates if either the trial participation or outcome model is correctly specified."

- **Limitations paragraph of Discussion section (Page 8, lines 329-332):**

"Sixth, our transportability analyses relied on several assumptions, including that all effect measure modifiers were measured and that models were correctly specified (see Methods). We conducted sensitivity analyses where possible, but violations of these assumptions cannot be ruled out."

3. I am not familiar with the specific context in Senegal, but can we be confident about the consistency assumption? In other words, could MDA implementation differ between trial and non-trial areas? If so, please clarify this point.

<u>Response:</u> We appreciate the opportunity to clarify our consistency assumption. The trial in Senegal was designed to be pragmatic trial, with MDA delivered as closely as possible to routine programmatic conditions. We clarify this point in the **Methods section on Page 15, lines 655-663):**

"[To make causal inferences about intervention effectiveness outside the original trial areas, several assumptions are required.]…Fifth, the consistency assumption states that the potential outcome under each intervention for any cluster is equal to the cluster's observed outcome, assuming that the intervention is implemented similarly in both trial and target settings. <u>We expect this assumption to hold as the trial was designed as a pragmatic trial with MDA delivered as closely to routine programmatic conditions as possible (i.e., via local community health workers).</u>"

4. Some graphical presentation might help readers understand the concept of transportability analysis.

Response: We have included an analytic flow diagram (**Figure 4**) to help readers understand the concept of transportability analyses.

Reviewer #2:

The paper describes models to predict the impact of MDA in populations living in the same region but their villages were not included in the trial. This is of value because MDAs can help in malaria control but are resource intensive. Being able to predict whether such an expensive intervention will be promising is today more important than ever before as funding of global health intervention is shrinking precipitously.

1. One of the most important variables predicting high impact of antimalarial MDAs is coverage. A highly transmissible organism like *P. falciparum* requires high coverage to make a lasting impact. Coverage is difficult to predict. Qualitative scientists have produced considerable amounts of research and still predictions are tricky. Within trials considerable variability has been documented between randomized villages. The authors do acknowledge the problem but have not included the unpredictability of coverage as a major limitation of their predictions.

Response: We agree with the reviewer about this point. We had considered alternative proxies for coverage as well, such as vaccine coverage of the Expanded Immunization Program in study areas, which may serve as a proxy for public health infrastructure operations and functionality. Unfortunately, the only data source we are aware of is the Demographic and Health Surveys, and the most recent year of data is from 2018. We felt that pre-pandemic coverage might not represent levels in 2020-2022, so we did not incorporate this data into our models. We did investigate predicted travel time to the nearest healthcare facility as another proxy for coverage, and **Appendix 14** shows a moderate correlation with coverage. However, we ultimately excluded travel time to health facility because model algorithms did not converge when it was included, possibly due to a lack of temporal variation.

We have revised the Limitations paragraph of our **Discussion section on Page 8, lines 315-324** to address this point:

"Fourth, our transported estimates assume that MDA would be delivered similarly in trial and non-trial areas and are intended to reflect intention-to-treat effects (i.e., the predicted effectiveness had non-trial areas been assigned to receive MDA). Because coverage varied across trial clusters, these estimates also rely on the assumption that coverage patterns in non-trial settings would resemble those observed in the trial. Our sensitivity analyses suggest that some of this variation was captured by measured effect modifiers (e.g., precipitation, daytime temperature; Appendix 14). However, as <u>coverage can also depend on other factors that are often difficult to predict (e.g., community acceptability and</u>

sensitization) our estimates may not fully account for this variation. It is also possible that coverage may be lower in real-world settings."

2. The authors write "travel time to health facility may serve as a proxy for MDA coverage in real-world implementation settings, and …". Travel time may play a role but it is far from a correlate. The reasons why people participate or do not participate in MDAs are complex. Reducing this complexity to travel time is an oversimplification. I understand the absence of alternatives but the authors should acknowledge this limitation.

Response: We agree and have provided more context for other correlates we considered in our response above. We have clearly noted this point in our Limitations paragraph (See underlined changes in response to Reviewer #2's comment #1)

3. Minor point. In Figure 3 the authors use the label 'c)' for two panels. Clearly one should be c) and the next d).

Response: This error has been corrected in **Figure 3**.

Reviewer #3:

A very well-written and methodologically rigorous manuscript that presents a novel application of doubly robust transportability methods to a cluster-randomized malaria trial in Senegal. The integration of ecological and demographic effect modifiers is commendable and contributes meaningfully to the literature on subnational targeting of malaria interventions, adding both operational relevance and policy utility.

To enhance transparency and interpretability, I recommend the following improvements (see below). Addressing these points would improve clarity and accessibility, and further elevate the manuscript's contribution to evidence-based malaria control.

1. Include model fit diagnostics to support the robustness of transported estimates.

Response: We have added **Appendix 17**, which includes model fit diagnostics for the trial selection model (Brier score) and the outcome model (Dispersion parameter and pseudo R-squared). Please see further details in our response to the subsequent comment.

2. While the modeling framework is clearly described, the manuscript does not report predictive accuracy metrics (e.g., RMSE, calibration plots). A brief summary or supplemental figure would strengthen confidence in the outcome models.

Response: We appreciate the reviewer's question about the predictive accuracy of our models. We have added **Appendix 17**, which includes details about the predictive accuracy of the elastic net models.

Our elastic net outcome model metrics are described in the table below, and we have added these to the manuscript as well (see additional details in the newly added **Appendix 17**). Our outcome model was for malaria cases, a count outcome, and so we calculated the mean absolute error (MAE) and pseudo R-squared instead of the RMSE. The MAE is more robust to the extreme values common in count data and pseudo R-squared provides an interpretable measure of model fit that accounts for the Poisson likelihood structure, whereas RMSE can be disproportionately influenced by outliers and doesn't account for the assumption that the variance equals the mean in Poisson models.

In the spirit of the reviewer's question, we explored a SuperLearner ensemble machine learning approach for the outcome model to assess if we could achieve our study objectives with a higher level of outcome model predictive accuracy. Candidate algorithms included generalized linear models, elastic net models, LASSO, ridge regression, generalized additive models, random forests and gradient boosting. Using SuperLearner, we were able to achieve a pseudo-R^2 close to 0.9.

	Elastic net regression		SuperLearner	
	Mean absolute error	Pseudo-R^2	Mean absolute error	Pseudo-R^2
Treatment arm model	4.1	0.42	2.0	0.89
Control arm model	7.6	0.44	3.5	0.91

However, we found that when we used SuperLearner to model our outcome, transportability estimates were unstable due to the relatively small number of units in the non-trial areas. In some cases, Commune-months with extreme predicted values had an outsize influence on the transported treatment effect estimates. It is likely that the ensemble machine learning approach achieved higher predictive accuracy by fitting complex patterns in the trial data, but the models extrapolated poorly to non-trial units with covariate patterns not well-represented in the trial. We also tested the SuperLearner approach in our validation analysis, where we recovered the treatment effect in the area immediately around the trial site. When stratified to the four Communes overlapping with the trial clusters (i.e., the four Communes included in the validation analysis), estimates from SuperLearner did not align with the trial's original effect estimate. In some cases, the predicted outcomes were 4 times the observed maximum cases, and the double robust estimator became numerically unstable due to large negative bias correction terms. This suggests that a high pseudo-R^2 within the trial did not guarantee valid transportability because the model extrapolated poorly. In contrast, as we reported in our original submission, when using elastic net models, the validation analysis aligned with the original trial's effect estimate, and the Commune-stratified analysis within the trial area aligned with the stratified effect estimate using trial data.

Thus, while it was valuable to explore using a SuperLearner approach for the outcome model, we believe our original approach is more appropriate given that our goal is to transport effects from the trial to target populations of interest rather than prediction within the trial sample. While elastic net models have a lower in-sample R^2 due to the smaller number of covariates contributing to the final prediction, they produced more

stable and plausible predictions given that the number of non-trial units was relatively small. Further, we were able to recover the treatment effect in the validation analysis and in the analysis of the Communes in the original trial using elastic net regression but not using SuperLearner.

3. The analytic pipeline spans multiple modeling steps but lacks a visual or tabular summary. A schematic or table outlining the workflow and model structure would improve reproducibility and help readers navigate the complexity.

Response: We have included **Figure 4**, which provides readers with a schematic of the analytic pipeline of our transportability analyses.

4. Although the causal assumptions underlying transportability are discussed, a concise summary table would make them easier to reference and evaluate, particularly for readers less familiar with causal inference frameworks.

Response: We appreciate this response and have added **Appendix 18** to outline the assumptions required for transported estimates to be interpreted as causal.
